# A Retrospective Consecutive Controlled Case Series Analysis of the Assessment and Treatment of Elopement in Children with Autism in an Inpatient Setting

**DOI:** 10.1007/s40617-024-00979-1

**Published:** 2024-10-24

**Authors:** Rose E. Nevill, Michelle F. Crawford, Jennifer R. Zarcone, Elizabeth Maquera, Griffin W. Rooker, Jonathan D. Schmidt

**Affiliations:** 1 Kennedy Krieger Institute and Johns Hopkins University School of Medicine, Baltimore, MD USA; 2 University of Virginia School of Education and Human Development, 417 Emmet Street South, Charlottesville, VA 22904 USA; 3 May Institute, Randolph, MA USA; 4 Mount St. Mary’s University, Emmitsburg, MD USA

**Keywords:** Elopement, Intervention, Neurodevelopmental disabilities, Safety, Single-subject design

## Abstract

This study included a retrospective consecutive controlled case series analysis of 17 function-based elopement treatments developed for 14 children with autism in an inpatient setting. Results from functional analyses indicated elopement was most frequently maintained by access to tangible items, followed by automatic reinforcement. All treatments used a combination of multiple consequence-based or antecedent- and consequence-based strategies. Individual treatment components and strategies for testing treatment generality are reviewed to provide examples of how such treatments may be implemented to reduce elopement attempts. Effective treatments, defined as reducing elopement by at least 80%, were developed for 13 children. These findings may help guide clinicians as they plan for the safe assessment and treatment of this difficult and dangerous behavior.

**Supplementary Information:**

The online version contains supplementary material available at 10.1007/s40617-024-00979-1.

Elopement, or leaving a specific area without permission or supervision, is a major safety concern for autistic people. Elopement presents risks of injury to those who elope as well as those who retrieve them, particularly when it occurs in the community. Epidemiological studies examining mortality rates among Americans with autism (Guan & Li, 2017a, 2018b) identified drowning as the third most frequent cause of death via accidental injury among children under 15 years; of those drowning cases, 74% were preceded by elopement. One analysis of Interactive Autism Network data found that 49% of parents of children with autism reported that their child had eloped at least once after the age of 4 (Anderson et al., 2012). Another analysis of nationally representative data (*n* = 1,420) from the Centers for Disease Control and Prevention’s (CDC) Survey of Pathways to Diagnosis and Services (CDC, 2011) found that 25% of parents of children with autism and 35% of parents of children with intellectual and developmental disabilities (IDD) including autism reported that their child had eloped over the preceding 12 months. Increased risk was associated with being younger (6–11 years old) and male. Children with autism without intellectual disability (ID) were more likely to elope if they had additional challenging behavior, emotional problems, and fewer self-care skills (Kiely et al., 2016).

The primary strategies recommended to address elopement risk in the general community are often restrictive and costly: using either electronic tracking devices or physical barriers (e.g., locks, alarms, gates; Kiely et al., 2016, 2018). These approaches have limitations in that they do not work to prevent attempts to elope or teach safe alternative behaviors; rather, they restrict opportunities to elope (CDC, 2011; Kiely et al., 2016). Further, one survey of 1,459 caregivers found that although the use of electronic tracking devices significantly reduced the frequency and risks of elopement, only 25% were current and 7% were past tracker users—respondents who were not using trackers reported that it was primarily due to the cost or their lack of awareness of such tools (McLaughlin et al., 2020). In addition, Pereira‐Smith et al. (2019) indicated that most families used antecedent strategies such as door locks and alarms, plus notifying neighbors of the risk as primary interventions, which, again, does not teach the individual safe alternatives.

The current standard for behavioral treatments targeting elopement, like other challenging behavior, is that they are informed by the results from a functional behavior assessment that identifies the situations that evoke elopement and the consequences that maintain it (O’Neill et al., 2014). In an ideal situation, functional behavior assessments include functional analyses (FAs; Iwata et al., 1982/1994a), in which the antecedents and consequences hypothesized to evoke and maintain elopement are directly manipulated in an experimental design to demonstrate a functional relation. However, due to safety issues related to assessing the function of a behavior that allows an individual to escape caregiver supervision, conducting FAs on elopement can be challenging. Thus, clinicians may turn to indirect assessments to determine the variables that give rise to and maintain elopement. Such assessments are limited in that they do not provide evidence of causal relations, are subject to faulty recall, and are not always accurate in identifying the function of challenging behavior (e.g., Rooker et al., 2015). For these reasons, reliance on indirect assessments may prove ineffective in assessing elopement to inform treatment.

There are additional challenges to completing successful treatment evaluations for elopement. The danger associated with elopement often requires caregivers to retrieve the person through chasing or response blocking. Such retrieval procedures deliver inadvertent attention, which may be particularly problematic for children whose elopement is maintained by positive reinforcement in the form of attention (Boyle et al., 2019). This means that specific treatment components (e.g., extinction) cannot be implemented with full integrity due to safety factors. Blocking the behavior can also be particularly challenging for caregivers to implement, especially when other children are present or the person is larger, faster, and/or more difficult to physically manage (Call et al., 2011). Despite these difficulties, there have been several single-case experimental design (SCED) studies that identified effective treatments for elopement, including increased supervision in the community or at school (Lang et al., 2010), reinforcement of appropriate walking or the absence of elopement (e.g., Piazza et al., 1997; Roane & DeRosa, 2014), functional communication training (FCT; Falcomata et al., 2010; Lehardy et al., 2013; Luczynski & Hanley, 2013; Stevenson et al., 2016), time-based access to reinforcement (Boyle & Adamson, 2017; Stevenson et al., 2016), noncontingent access to preferred items that initially motivated elopement (e.g., Lang et al., 2010; Piazza et al., 1997; Tarbox et al., 2003), and response blocking (Call et al., 2011).

To date, there have only been two large-*n* studies examining the effects of behavioral treatments for elopement in children with ASD. The first randomized controlled treatment evaluation for elopement (Scheithauer et al., 2020) used parent education and behavioral skills training to teach parents (*n* = 24) to collect data, identify the function of elopement through a trial-based FA, and implement a set of behavioral treatments (i.e., differential reinforcement [DR], extinction, noncontingent reinforcement, and response cost). Significant reductions in elopement were found for 42% of participants in the active treatment group versus only 17% in the control group. Although these results are promising, direct measures of elopement were not collected; rather, elopement was assessed based on clinician coding of parent reports using the Clinical Global Impressions Scale (Busner & Targum, 2007) and other parent rating scales, including the Aberrant Behavior Checklist (Aman et al., 1985) and the Home Elopement Safety Checklist [Scheithauer et al., 2020]). The second study was a retrospective chart review of 11 patients who received behavioral treatments for elopement (Call et al., 2017). This study reported summary data of percentage reduction in elopement based on direct observation. It also showed behavioral treatments were effective in reducing elopement attempts, with large treatment effects (Cohen’s *d* = 1.18). However, because this was a brief report of the treatments, the authors only listed the treatment components; they did not provide details on how the treatments were arranged, the types of alternative reinforcers used within the treatments, or how the effects were evaluated.

Although there is established evidence for the efficacy of behavioral treatments on elopement, there is a lack of description of the treatment components and details on the use of SCED from large-scale studies that can be used to generate recommendations for effective treatment selection based on topography, function, or intensity of elopement. An essential next step would be to examine differential outcomes based on treatment or child characteristics, such as treatment components used or functions identified. A consecutive controlled case series (CCCS) is an exploratory method that examines within and across-treatment outcomes for cases implementing SCEDs that share a common characteristic and are completed consecutively within a given time frame. They offer the advantage of presenting case-level data for treatments that target a similar function, topography of challenging behavior, or evaluate a shared treatment approach regardless of the outcome of each treatment, thereby minimizing treatment or publication bias (Hagopian, 2020). This is an advantage on previous larger scale studies of elopement interventions such as Call et al. (2017), which reported summary data for percentage reduction only. Although findings from SCEDs, by nature, cannot be generalized to broader populations, methods such as CCCS that result in summary data facilitate the generality of findings across larger populations with shared characteristics or who are receiving the same treatments. Due to the dire consequences of elopement, studies such as CCCS are needed to help guide clinicians in selecting evidence-based treatments for elopement.

The current study conducted a retrospective CCCS analysis of patients seen for the assessment and treatment of elopement through an intensive inpatient unit for severe challenging behavior. Like Call et al. (2017), the effects of behavioral treatment packages that directly targeted reductions in frequencies of elopement attempts and the extent to which behavioral treatments reduced elopement to consistent rates of zero or near zero levels were explored. Treatment packages that demonstrated experimental control over elopement using a SCED were included to ensure the validity of outcomes. To expand the existing evidence base for elopement treatments, records of identified functions of elopement and treatment components used were reviewed and summarized.

## Method

### Search Procedure

**Fig. 1:**
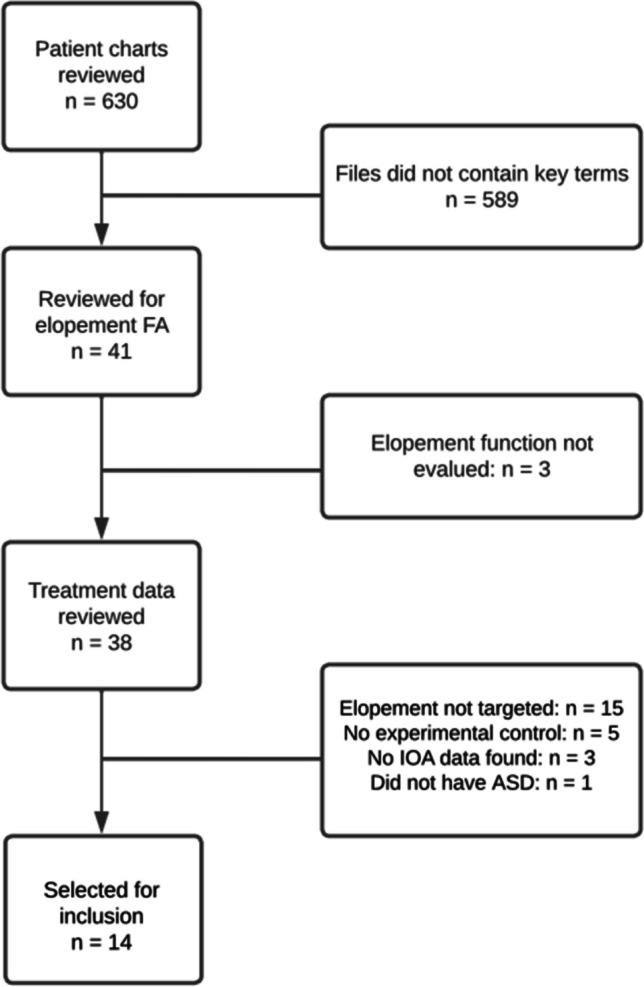
Participant Inclusion Flowchart

**Fig. 2:**
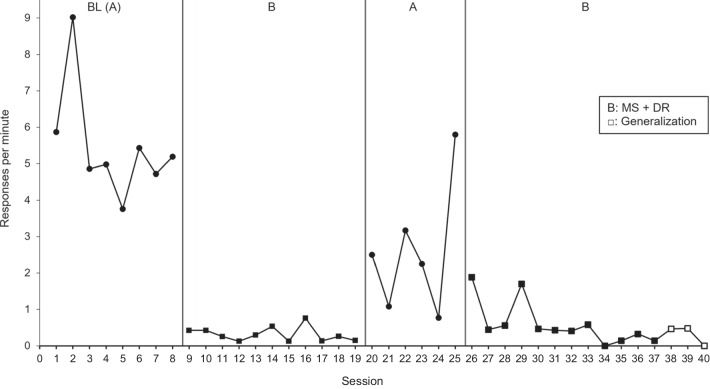
Treatment 1 Evaluation

**Fig. 3:**
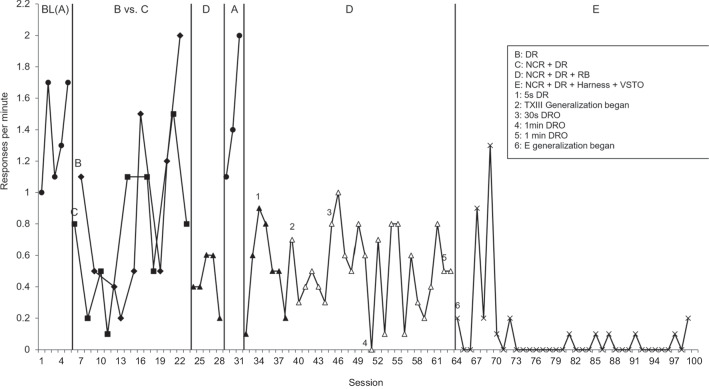
Treatment 2 Evaluation

Electronic data records from the inpatient unit, available for all patients admitted between 1997 and 2020 (*n* = 630), were reviewed to identify patients who received a functional analysis targeting elopement as a behavior of clinical concern during their admission. The following keywords were used to search the patient record database: “elop” (to be inclusive of all variations of the word elopement), “run,” “bolt,” and “AWOL.” Participant inclusion criteria were: (1) having received an FA targeting elopement; (2) having received a treatment evaluation for elopement, regardless of treatment effects; (3) a SCED (e.g., a reversal or multiple baseline design) was used that could permit the demonstration of functional control, regardless of whether it was truly demonstrated; (4) interobserver agreement data (IOA) being available for a minimum of 20% of treatment sessions; and (5) having a diagnosis of autism. The search identified 41 individual patient cases where elopement was reported to be a target behavior. Application of the inclusion criteria resulted in a final sample of 14 individuals with a treatment targeting elopement (see Fig. 1 for a Participant Inclusion Flowchart with reasons for exclusion). Two treatment evaluations were completed for Participant 11 (P11) and three for P14 to target individual functions identified within FAs. This resulted in 17 treatment applications across 14 participants (Figs. 2 and 3 show graphical outcomes for P1-2, Supplemental Figs. 4–16 show graphical outcomes for P3-14). Treatment outcomes for P10 (Fig. 11) were also reported in Frank-Crawford et al. (2024).^1^ For clarity, participants’ treatments will be referred to using their participant number (i.e., Tx 1–14), P11’s two treatments will be referred to as Txs 11(1) and 11(2), and P14’s three treatments will be referred to as Txs 14(1), 14(2), and 14(3).

### Participants

Participants were 14 children (13 males, 1 female) aged 5- to 21-years-old (*M* = 11.43, *SD* = 4.24) who were admitted for short-term treatment in an inpatient hospital unit for the assessment and treatment of severe challenging behavior displayed by individuals with IDD. Participants were typically referred for the assessment and treatment of multiple topographies of challenging behavior (e.g., aggression, self-injury) in addition to elopement, and were admitted for 3 to 8 months (*M* = 4.86 months, *SD* = 1.61). All participants had a previous diagnosis of autism and ID. A review of clinical records found that two had mild, three had moderate, four had severe, two had profound, and three had an unspecified ID (see Table 1). Although all participants received FAs and treatment evaluations for multiple topographies of challenging behavior, the present study reports only on the FA and treatment outcomes for elopement.

**Table 1:** Participant Demographics, Adverse Outcomes Due to Elopement, and Elopement FA Results

				Elopement FA Outcomes		
Case	Age	ID Severity	Adverse Outcomes due to Elopement	Function Identified	Control (RPM)	Test (RPM)
1	12	Moderate	Restricted community access	Auto	4.67	6.02
2	10	Profound	Restricted community access	Auto	1.32	1.30
3	10	Profound	History of jumping out of moving cars, found in strangers’ cars, restricted community access	Auto	0.82	0.72
4	16	Severe	Locked windows and doors at home, restricted community access	Auto	0.00	0.55
5	10	Moderate	Not referenced	Tan	0.82	2.02
6	15	Moderate	Locked windows and doors at home and school	Tan	0.13	0.64
7	5	Severe	Restricted community access	Tan	0.00	1.30
8	9	Mild	Broken pelvis after being hit by a car, restricted community access	Tan	0.00	0.62
9	21	Unspecified	Restricted community access	Tan	0.00	2.08
10	6	Unspecified	CPS reports, conflict with neighbors, locked windows and doors, home modifications to restrict exiting, restricted community access	Tan	0.00	1.50
11	17	Severe	Not referenced	Tan, Attn	0.00	Tan: 0.68, Attn: 0.88
12	11	Severe	Restricted community access	Auto, Tan	0.16	Auto: 0.27, Tan: 0.83
13	9	Unspecified	Locked windows and doors, police searches, restricted community access	Auto, Tan, Attn	0.06	Auto: 0.15, Tan: 0.98, Attn: 0.20
14	9	Mild	Found on highways, in neighbors’ homes, and strangers’ cars; broken foot; restricted community access	Tan-O, Tan-A, Attn	0.00	Tan-O: 1.12, Tan-A: 1.26 Attn: 0.82

Attn = Attention; Auto = Automatic, Esc = Escape; FA = Functional Analysis, ID = Intellectual Disability, RPM = Responses per minute, Tan = Tangible (O = Object-focused, A = Activity focused)

### Setting

#### Assessments and Treatment Evaluations

The locations of assessments and treatment evaluations were chosen to facilitate opportunities to elope safely while minimizing the necessity to physically block elopement. Assessments were conducted in the same settings as treatment evaluations for P1, 3–5, 13, and 14, and in different settings for the remaining participants. Sessions with P1 were conducted in a large auditorium with closed, unlocked doors, allowing opportunities to safely elope from the treatment team without requiring physical intervention. Sessions for P3 were conducted in a classroom located off the unit that presented the opportunity to elope safely into a connected, enclosed room. Sessions were conducted in a conjoined session room for Ps 4–5, 13, and 14.

The FAs for P2 and P8–10 were conducted in a large open hallway on the treatment unit with a free-standing mat at the end of the hallway to create a visual barrier and prevent other patients from entering or exiting the area. Most treatment evaluation sessions for P2 and P12 were conducted off the hospital unit (see *Generality Sessions*). Treatment evaluations for Ps 8–10 were completed while walking around the unit without any mats obstructing their path, and as many doors as possible were open to allow increased opportunities to elope. P6. Assessments for P7 were completed in a conjoined treatment room, whereas the treatment evaluation was completed by walking around the unit with bedroom and activity area doors open. All sessions for P11 were conducted in a large open room that contained a couch, chairs, and a small table.

#### Generality Sessions

Generality of treatment effects were tested by moving treatment evaluation sessions from the original testing area to other areas on the hospital unit, new adults, application with parents, and out into the community (i.e., surrounding hospital public spaces, parks, restaurants, trips home, and stores). Generalization processes varied across treatments as a function of length of hospital duration and availability of parents to implement sessions. Generalization of Tx 2 began early after major initial reductions in elopement attempts were observed, though rates were continuing to vary between 0.1 and 0.9 rpm. Generalization sessions were conducted in a contained open tunnel system in the associated hospital, and then broadened to hospital hallways, cafeterias, courtyards, on community outings, and with parents once rates became more stable. All treatment evaluation sessions for Tx 12 were completed outside the treatment unit in surrounding buildings and the community to address elopement specifically in the context of transitioning. Generalization of Txs 1, 6, 8, 13, 14(2), and 14(3) was evaluated in the community (i.e., stores, park, restaurants) only after low, stable rates of elopement were observed in treatment sessions on the inpatient unit. Txs 3, 4, 5, 7, 9, 10, 11(1) and 11(2) were transitioned out of the initial treatment room or unit hallway in which the treatment evaluation was initiated, into common areas of the hospital unit. One generalization session was completed at the conclusion of the evaluation for Tx 14(1) to determine if elopement would occur in the community because it did not occur in the hospital unit. Generalization of Txs 14(2) and 14(3) was conducted with P14’s mother in the community and was scheduled based on when the mother’s availability to conduct sessions.

#### Measures

*Elopement* was generally defined as moving more than 3 ft from a caregiver without permission or leaving a confined area without a caregiver, consistent with previous definitions in the elopement literature (e.g., Lang et al., 2010). Definitions for P1, 4, 5, 6, and 14 included more specific qualifiers to their operational definition. Elopement was not recorded for P1 unless it occurred after 5 s walking independently beside the therapist. For P4, elopement also included attempting to or successfully opening the door to a room by grabbing the door handle. For P5 and P14, elopement was also scored when they placed at least 1 ft past the threshold of the door to exit the room in which all sessions occurred. For P6, elopement was defined differently across contexts. In an open area (e.g., hallway), elopement was defined as moving beyond 3 ft from a therapist. In a closed area (e.g., a room), elopement was defined as attempting to or successfully running towards or going beyond an open door. *Rate*, defined as elopement attempts or successes per minute, was the primary dependent variable across treatments except Tx 10, which tracked latency to elopement (the duration from session start to elopement attempt) and rate. To facilitate comparison across treatments, all data are reported as rates. We assessed the number of treatments that achieved an 80% or greater reduction in elopement, the inpatient unit’s standard minimum goal for challenging behavior reduction. In addition, treatment evaluations were conducted over the timeframe of a patient’s hospital admission, and discharge dates often prevented opportunities for treatment evaluations to continue until a 100% reduction was achieved.

### Procedures

#### Data Collection and Interobserver Agreement

Data from FAs and treatment evaluations were collected using BDataPro software (Bullock et al., 2017) on laptop computers or using paper data sheets. Data from all generalization sessions were collected using paper data sheets.

All assessments and treatments were implemented by behavioral treatment teams, comprised of one primary therapist and two backup therapists, under the direct, daily supervision of faculty-level board certified behavior analysts (BCBAs) with master’s or doctoral-level training in behavior analysis and extensive experience in the assessment and treatment of severe challenging behavior in the IDD population. Thus, all assessment and treatment components were selected based on the discretion and expertise of the supervising BCBAs and in consultation with the caregivers for each participant. All team members had intensive training in core treatment procedures (e.g., extinction/planned ignoring, least-to-most prompting, response blocking, DR), which were standardized by unit-wide protocols.

To promote high IOA, secondary observers were required to be trained to a 90% or higher agreement criterion with primary observers during FAs, treatment evaluations, and generalization sessions. IOA was collected for a minimum of 20% of sessions for all participants (*M* = 39.89, range = 20.00%–63.00%). The mean exact IOA of elopement per minute, across participants, was 96.8% (range = 91.81%–100%).

#### Functional Analysis

FAs were conducted using multielement (*n* = 12), reversal (*n* = 1), or combined reversal/multielement (*n* = 1) designs. All FAs measured rpm of elopement except for Tx 10, which measured latency to elopement after session start. Sessions were 5 or 10 min in duration. Across FAs, elopement received programmed contingencies, aggression and self-injury were blocked in the absence of verbal attention, and all other challenging behaviors were ignored. Conditions were selected for inclusion based on information gathered from intake paperwork and open-ended behavioral interviews completed with the parents by the BCBA at the time of admission. Within the FA, at least two test conditions were compared to one control (toy play) condition: attention, demand, ignore, alone, and tangible.

In the *attention condition*, therapists diverted their attention from the participant, and contingent on elopement, attention was provided in the form of verbal reprimands without response blocking for 30 s. In the *tangible condition*, the therapist and participant were present in an area with preferred items or activities out of the participants’ reach by at least 3 ft (Txs 1–2, 8–11) or in a connected room (Txs 3–7, 12–14) but within their line of sight. Contingent on elopement to the item or activity, the participant received 30 s access before being prompted to stop interacting with the item. In the *demand condition*, the therapist presented task demands (i.e., schoolwork or chores) to the participant using least-to-most prompting. Contingent on elopement, the therapist removed the task and ignored the participant for 30 s before presenting a new demand. The 30 s reinforcement interval for all social-reinforcement test conditions was removed from the total session time in calculating the rate of elopement. For the tangible and demand conditions, resetting procedures were implemented following reinforcement that consisted of using least-to-most prompting to guide participants back to the original location, except for P1 and P11 whose FAs were conducted in large open areas where items were re-presented to the participant in the location they were in following reinforcement. Resetting intervals were also excluded from rate calculations. In the *ignore condition*, the therapist was present in the area with the participant but ignored the elopement. The *alone condition* was implemented when the supervising BCBA considered it safe to leave the participant alone in a room to observe whether elopement continued when no social contingencies were in place. In the *control (toy play) condition*, the therapist and participant were present in the same area, the participant had continuous access to preferred items, activities, and the therapist’s attention, the therapist did not place any demands on the participant, and elopement was ignored.

One specific modification worth noting occurred in P3’s FA, which conducted all five tested conditions (attention, tangible, demand, ignore, toy play) in a divided room with two chairs in each section. In this FA, a tone was played every 40 s to signal to the therapist to physically guide P3 to sit in a chair on Side A. If P3 eloped to Side B, or continued to move out of the room, he was physically guided back to Side A to sit in a chair at the sound of the tone. If P3 was already seated in a chair on Side A, or had returned to Side A independently, he was physically guided to sit in a different chair on Side A at the sound of the tone. This procedure was used to control for the effect of physical attention across conditions.

A minimum of three series of all included conditions were completed for each participant, and sessions continued until clear differentiated responding was observed. The function of elopement was determined by supervising BCBAs using visual inspection procedures as outlined by Hagopian et al. (1997) or Roane et al. (2013) when fewer than 10 data points were available (see Table 1). For an exemplar FA graph from P7, see supplemental materials Fig. 17.

#### Competing Stimulus Assessment

A competing stimulus assessment (CSA; Ahearn et al., 2005; Fisher et al., 2000; for a review, see Haddock & Hagopian, 2020) was conducted with Ps 2, 3, 4, 6, 9, 10,12, and 13 to identify objects (i.e., toys, tablets, sensory items) that produced high levels of engagement and low rates of elopement. CSAs measured latency to elopement for P10 and rpm for all remaining participants. Items were selected for inclusion in the CSA based on behavioral observation, parent interviews, and clinical record review. One item was presented per session in a multielement design and levels of elopement were compared with a control condition in which no stimuli were present, except for P3 who was exposed to items in a pairwise format. Sessions were 5 or 10 min in duration, and the contingencies for elopement were the same as those used in the FA condition that produced differentiated levels of elopement relative to the control.

P6 received two CSAs: one to identify competing items while performing seated activities and a second to identify competing items during transitions. In the CSAs for P6 and P13, after observing low engagement rates during initial sessions, adult attention was provided contingent on the absence of elopement, which resulted in higher rates of engagement with competing stimuli. Synchronous attention was also provided for P2 but did not have a differential effect on engagement with competing items. Therefore, edible reinforcers were delivered every 5 s without an elopement attempt, and engagement with competing stimuli increased. Ultimately, 1 to 3 items identified through CSAs were added to subsequent treatment evaluations across participants.

#### Stimulus Avoidance Assessment

A stimulus avoidance assessment (Fisher et al., 1994) was conducted with Ps 2, 3, and 12. Prior to implementing the assessment, procedures were described to parents in detail, and consent was obtained. Parents were requested to complete a social validity rating scale to identify any procedures they would not find acceptable, which were then excluded. Procedures potentially assessed included a baskethold, a chair time-out, a visual screen time-out, contingent demands, and hands down (see Fisher et al., 1994, for a description of these procedures). Procedures approved for testing were presented across conditions in a multielement design. Sessions were 10 min and video recorded to ensure appropriate oversight and reliability coding. Contingencies mimicked those in the ignore condition from an FA, except that the targeted procedure was implemented noncontingently every 1 min for 30 s. To avoid adventitious reinforcement, the procedure was delayed by 5 s if the participant attempted to interact with the therapist. Following each session, participants were provided a 2 min break with preferred activities. The duration of negative vocalizations and avoidant behaviors, and frequency of elopement attempts, were recorded as dependent variables. After completing the assessment, a procedure was selected for inclusion in the treatment package if it was deemed socially acceptable by the patient’s family and was likely to reduce elopement based on rate of elopement attempts recorded during individual sessions.

#### Treatment Evaluations

##### Experimental designs

Each participant received an individualized treatment evaluation for elopement. Of the treatment evaluations that were included, the following designs were implemented: 10 used a reversal (Txs 1, 4, 5, 7–13), two (Txs 2, 3) used a reversal with an embedded multielement design, one (Tx 6) used a multiple baseline across reinforcement types, and three (Txs 14[1–3]) were compared through a multiple baseline across functions design. A minimum of three sessions were conducted during baseline, in which elopement received reinforcement from the therapist conducting the evaluation if a social function was identified, or no contingencies when an automatic function was identified. Once the treatment was introduced, a minimum of two sessions were conducted before reversing back to baseline or introducing a new treatment condition. Session duration started at 5 min for seven treatments (Txs 4, 5, 7, 10, 11[1], 11[2], 13) and 10 min for 10 treatments (Txs 1–3, 6, 8, 9, 12, 14[1]–[3]). Case records indicated that session duration was extended for Txs 4 (15 min), 5 (5.5 min), 11(1) and 11(2) (30 min), and 8, though the final duration for Tx 8 was not reported.

##### Treatment components

Assessment results (e.g., FAs, CSA) and evidence from the behavior-analytic elopement literature were used to select treatment components. Antecedent and consequence-based procedures were used across treatments. Treatment components were added incrementally, starting with antecedent or antecedent and reinforcement-based procedures in 14 treatments and progressing to add additional treatment components if the initial treatment did not result in an 80% reduction in elopement. The exceptions to this were Txs 9, 10, and 12, which included response blocking in the initial treatment phase, which was faded out in the final treatment phase of Txs 9 and 12. In some cases, punishment procedures were added after antecedent and punishment procedures alone did not yield an 80% reduction in elopement. Punishment procedures had to be implemented by following clear pre-written implementation guidelines that were standardized for the hospital unit, and their use was closely monitored by supervisors. Individual punishment procedures are described below. Descriptions of individual components and how they were used are included below. For a tabular display of treatment components included in final treatment packages, see Table 2.

**Table 2:** Individual Intervention Components included in Final Treatment Packages

	Treatment and Function Targeted																
Treatment Component (Frequency of use)	1 (AU)	2 (AU)	3 (AU)	4 (AU)	5 (T)	6 (T)	7 (T)	8 (T)	9 (T)	10 (T)	11(1) (T)	11(2) (AT)	12 (M)	13 (M)	14(1) (AT)	14(2) (T-O)	14(3) (T-A)
*Antecedent-based*																	
NCR (7)		X							X	X	X	X		X		X	
Multiple schedules (8)	X				X		X			X	X	X				X	X
Hand holding (5)							X	X	X	X			X				
*Behavior-promoting*																	
DR (7)	X	X	X	X	X			X					X				
Chained schedules (2)			X	X													
*Instructional Methods*																	
FCT (10)			X	X		X	X			X		X		X	X	X	X
Social Skills Teaching (1)																	X
*Behavior-reductive*																	
Extinction (10)				X		X	X			X	X	X		X	X	X	X
Response blocking (4)		X								X	X	X					
Baskethold (1)			X														
Visual Screen Time-Out (2)		X											X				

*A*, Activity; *AU*, Automatic; *AT*, Attention; *DR*, Differential reinforcement; *FCT*, Functional Communication Teaching; *M*, Multiple; *NCR*, Noncontingent Reinforcement; *T*, Tangible; *O*, Object

##### Noncontingent reinforcement

Noncontingent reinforcement (NCR) was implemented as a treatment component for one treatment targeting an automatic function, four targeting tangible functions, one targeting an attention function, and one targeting multiple functions. Across treatments, NCR was delayed by 5 s if elopement occurred to avoid adventitious reinforcement. NCR involved a choice of multiple competing items identified through a CSA, on a fixed-time (FT) schedule. Items were available noncontingently while the participant was walking with the therapist (Txs 2, 9, 13) or while spending time in a large open space (Txs 11[1], 11[2]). NCR with moderately preferred items was used in Txs 10 and 14(2) to help participants tolerate when highly preferred items were unavailable. NCR schedules were faded in Tx 9 from FT-10 s to FT-30 s.

##### Hand Holding

Therapists would lightly hold the participant’s hand in Txs 7–10 and 12 while walking. In these instances, hand holding was not used as a form of response blocking, and treatment documentation noted that contingent on an attempt to pull away their hand, the therapist would let go. Hand holding was included in all treatment phases of Txs 7, 8, 10, and 12. Tx 9 added hand holding in the final treatment phase after finding that noncontingent reinforcement alone did not result in sufficient response reduction.

##### Differential reinforcement

DR was used in four treatments targeting an automatic function, two targeting a tangible function, and one targeting multiple functions. DR with a resetting interval was used for walking within arm’s reach of the therapist (Txs 1, 2, 8, 12) or waiting for a set period (Tx 5). Compliance with demands (i.e., schoolwork, chores) without elopement was reinforced with a token in Txs 3 and 4. The fixed interval DR schedules were thinned systematically to increase the interval of time participants were required to walk safely without eloping to receive reinforcement in Txs 2 (up to 1 min) and 8 (up to 5 min), as was the fixed ratio DR schedule for Tx 3 (continuous reinforcement to an FR10). A choice of reinforcers was provided in all treatments except Txs 8 and 12, where a single edible reinforcer was presented that was identified as the most preferred snack through a preference assessment.

##### Multiple schedules

Multiple schedules of reinforcement were used in one treatment targeting an automatic function, six targeting a tangible function, and one targeting an attention function. Components of the multiple schedules were signaled using a stimulus card (e.g., red/green) worn on a lanyard by a therapist while transitioning (Txs 1, 10). Txs 7, 11(1), 11(2), 14(2), and 14(3) used red/green board presented on a table during seated activities. Reinforcers were available contingent on a communicative response in the presence of the green stimulus and unavailable in the presence of the red stimulus and the contingencies were explained verbally at the start of each session. In Txs 1 and 5, a red/green card was used to signal that a resetting DR of walking within arm’s reach of the therapist (green, Tx 1) that was reinforced with a 20-s break (red, Tx 1), or waiting in a room without access to a preferred reinforcer (red, Tx 5) that was reinforced with access to the reinforcer (green, Tx 5). Tx 10 used a red/green board to signal the availability of opportunities to look at a specific object of interest. Requests to look at the object were honored when a picture icon representing the object was placed on the green side and ignored when it was on the red side.

Schedule thinning was implemented in Txs 7, 11(1), 11(2), 14(2), and 14(3) to increase the amount of time participants could tolerate the unavailability of reinforcers. Elopement attempts needed to be reduced by a minimum of 80% for at least one session to meet criteria for thinning to the next level across treatments and was decided at the discretion of the supervising BCBA. The duration of the extinction components was increased to 2 min in Tx 7, 10 min in Tx 11(1), 9 min in Tx 11(2), and 5 min in Txs 14(2) and 14(3).

##### Chained schedules

Chained schedules of reinforcement were used in two treatments targeting automatic functions. In Tx 3, if P3 placed tokens earned through DR of academic task completion, as described above, on a token board and handed it to the therapist, he received a small piece of food. This contingency was kept in place after introducing a choice in reinforcement and the choice to request his reinforcer using a functional communication card (see above). In Tx 4, the completion of an individual work task (e.g., matching a letter) was reinforced continuously with a token being placed on P4’s token board. Once P4’s token board had been completed twice, compliance without reinforcement was reinforced with a break for the rest of the session. Once consistent task engagement in the absence of elopement was observed, token reinforcement was thinned to an FR5 schedule and sessions were increased from 10 to 15 min. After successfully maintaining low rates of challenging behavior for an additional 10 sessions, the response requirement was increased to an FR 15 schedule, and the reinforcement period was increased to 5 min.

##### Extinction

Extinction was implemented in one treatment targeting an automatic function, six targeting tangible functions, two targeting attention functions, and one targeting multiple functions. Across all treatments, therapists actively observed to monitor and ensure safety while refraining from commenting on the behavior or showing an emotional reaction. In most cases, extinction was used in combination with an antecedent-based treatment. Extinction was trialed in Txs 3 and 12 but then replaced with a punishment procedure.

##### Functional communication training

FCT was conducted to teach the participant a communicative response in five treatments targeting a tangible function, two targeting an attention function, and one targeting multiple functions. Two treatments targeting an automatic function also incorporated general communication training, in which participants were taught a communication response to access competing items. Participants were most frequently taught to hand a picture card to the therapist to gain 30 s access to a preferred item (Txs 6, 7, 14(1)–14(3)) or attention (Txs 4, 11(2). Different approaches were used in Txs 10 and 13, where the reinforcer motivating elopement varied. In Tx 10, an omnibus mand to “go see” something in the environment was taught. In Tx 13, an initial attending response was taught to P13 to request therapist attention and access their communication device, which he then used to request reinforcers. Later, P13 kept the communication device by wearing it on a strap. Across all treatments that included FCT, a least-to-most prompting hierarchy was used to teach the communication response which occurred before adding FCT into elopement treatment evaluations to ensure mastery.

##### Delay tolerance training

In Tx 10, once a low and stable rate of elopement was established following FCT, a delay tolerance was introduced to teach P10 to tolerate waiting for reinforcement for up to 5 min. Then, firm denials of functional communicative requests to “go see” were introduced on a variable basis to prepare P10 for times in their natural environment when reinforcement would be unavailable.

##### Social skills teaching

Social skills teaching was used in one treatment targeting a tangible function, specifically access to preferred ways of playing activities. It was introduced with P14 before the third treatment phase to increase tolerance for not being able control how activities were played. Specific skills targeted were sharing with others, waiting for a turn, and accepting others’ ideas and behavioral skills teaching (modeling, rehearsal, role play with practice, and feedback; Parsons et al., 2013) was the instructional approach used. Once P14 successfully demonstrated targeted skills in over 90% of trials, this treatment component was incorporated into Tx 14(3).

##### Response blocking and harness use

Response blocking was included in one final treatment package targeting an automatic function, two targeting tangible functions, and one targeting an attention function. In Txs 2, 11(1), and 11(2), NCR and reinforcement-based procedures were trialed before introducing response blocking. In Tx 2, the treatment team introduced response blocking during the third treatment phase which produced modest decreases in elopement. Therefore, a harness with a long tether to limit how far the participant could elope prior to stopping the behavior replaced response blocking in the final treatment phase. Txs 9 and 12 began with the use of response blocking, but these were removed once consistent low rates of elopement were observed. In Tx 9, response blocking was introduced in the first treatment phase given that P9 was a large, 21-year-old adult whose elopement presented even higher safety concerns to self and others. Once stable and low levels of elopement were observed, response blocking was briefly removed, resulting in an increasing trend in elopement. In the third treatment phase, a prompt to hold an adult’s hand was added, with the adult letting go of P9’s hand contingent on an elopement attempt. This was used in combination with NCR to mitigate elopement attempts. Response blocking was included in the initial phase of Txs 10 and 12 to be consistent with behavioral procedures that had been occurring at home and school prior to admission. Response blocking remained in place for Tx 10, and in Tx 12, response blocking was replaced with an empirically identified punishment procedure (i.e., visual-screen time-out).

##### Baskethold

Following highly variable rates of elopement in four previous treatment phases employing reinforcement-based procedures, a 30-s baskethold was added during the final treatment phase for P3 targeting an automatic function. Here, a baskethold was implemented for 30 s by one or two therapists, after which P3 was prompted to return to the previous activity. The baskethold procedure remained in place as P3 was discharged. Baskethold procedures involved the therapist grabbing the participant’s forearms and crossing their arms in front of their own body, in a seated or standing position, to prevent elopement.

##### Visual screen time-out

A visual screen time-out, in which the therapist briefly held their hand or a screen (e.g., card) in front of the participant’s face but without touching the participant, to obstruct their view, was added to the final treatment phase of one treatment targeting an automatic function and one treatment targeting multiple functions. In Tx 2, three previous treatments were trialed relying on reinforcement procedures, with the third treatment phase also including response blocking. In the fourth treatment phase, a 30-s duration visual screen time-out was added after generalizing the treatment into the community and an increase in elopement attempts was observed. It was added into Tx 12 for a 1-min duration following one previous treatment phase that involved reinforcement-based procedures, hand holding, and response blocking. These procedures were initiated with the intent to phase out the number of punishment procedures included in the treatment package, however P12 was discharged prematurely before fading could be initiated.

#### Procedures for Testing Generality of Treatment Effects

Given the inherent concerns related to generality of treatment effects stemming from procedures implemented in a highly controlled setting, treatment teams followed several procedures to maximize opportunities to test for generality. First, lead therapists held weekly update calls with primary caregivers to describe current procedures being evaluated and gain their feedback on the feasibility of and buy-in for implementing these treatment procedures at home and in the community. Once consistently low rates of responding were observed and treatment packages were finalized, teams conducted generality sessions. Overall, generality sessions occurred in different settings (e.g., the community), and procedures involved a primary therapist running the session and one to two backup therapists who had previously established IOA with the lead therapist observing and collecting data using paper-based data collection sheets. Once treatment evaluations successfully reduced elopement attempts by 80% or greater and resulted in consistent, low rates of elopement, these same treatments were implemented by other therapists, direct care staff, and caregivers in the inpatient unit. Final treatment packages were also taught to community-based behavior therapists and teachers, if available. Supervising behavior analysts provided guidance to community-based treatment teams and families around systematic fading procedures to implement once consistent low rates of elopement were observed following discharge.

#### Case and Data Analysis

To characterize the sample, qualitative and quantitative data were collected from participants’ admission records regarding adverse outcomes associated with participants’ elopement (e.g., injuries, restrictions) prior to their admission. FA graphs were reviewed to determine functions of elopement identified by treatment teams. Authors reexamined FA graphs independently applying the Hagopian et al. (1997) or Roane et al. (2013) criteria and were in 100% agreement with identified functions across participants. To examine treatment evaluation outcomes, authors calculated the overall rate of elopement reduction for each individual treatment phase relative to baseline. To summarize elopement treatment effects across participants, the average response reduction was calculated across all treatment packages and for treatments grouped by function of elopement. Furthermore, for cases where treatment teams tested generality of treatment effects, clinical records were reviewed to characterize the location of sessions and types of caregivers involved, number of sessions conducted, and mean elopement rates across these sessions.

## Results

### Adverse Outcomes Due to Elopement

Records of adverse outcomes associated with elopement prior to admission were available for 12 participants. All required constant supervision and had restricted community access. Two were reported as having jumped from moving vehicles, two had been struck by a car and sustained broken bones, one participant required police intervention to locate him after eloping, and one participant’s family was investigated by child protective services due to reports from concerned neighbors. Two participants had been found in strangers’ cars and neighbors’ homes following elopement episodes (see Table 1).

### FA Outcomes

Elopement was maintained by tangible reinforcement for six participants and automatic reinforcement for four participants. Four participants demonstrated elopement with multiple functions, although a tangible function was identified for all multiply maintained cases. P11 eloped to access tangible and social positive reinforcement, P12 eloped to access automatic and tangible reinforcement, P13 eloped to access automatic, tangible, and social positive reinforcement, and P14 eloped to access preferred objects (tangible–objects), preferred ways of play (tangible–activities), and attention. Across participants, mean rates of elopement were 1.20 responses per min (rpm; range = 0.15–6.02) in the test condition of identified functions and 0.57 rpm (range = 0.00–4.67) in the control condition. FAs that identified an automatic function as at least one functional reinforcer measured 1.50 rpm on average in the test condition (range = 0.15–6.02) and 1.17 rpm on average in the control (range = 0.00–4.67). An automatic function was demonstrated by high rates of elopement across all conditions tested with P1 (attention, escape, control), P2 (attention, escape, ignore, tangible, control), and P3 (attention, escape, ignore, control); and by zero attempts in the control compared to a high rate in the ignore condition for P4. FAs that identified a tangible function as at least one functional reinforcer measured an average 1.18 rpm in the test (range = 0.62–2.08) and 0.12 average rpm in the control condition (range = 0.00–0.82). FAs that identified a social positive function as at least one functional reinforcer measured an 0.63 average rpm in the test (range = 0.20–0.82) and 0.02 rpm in the control condition (range = 0.00–0.06). See Table 1 for mean elopement rates observed in control and test conditions of identified functions for individual participants.

### Treatment Evaluation Outcomes

Table 3 provides a summary of treatment evaluation data, including elopement rates at treatment baseline, percentage reduction from baseline for each subsequent treatment phase, and individual treatment components included in each treatment phase. The mean rate of elopement across all 17 treatments was 1.24 rpm at baseline. Mean rate at treatment endpoint across all treatments was 0.08 rpm, resulting in an overall 93.27% reduction. Txs 14(1) and 14(2) demonstrated zero or close to zero rates in baseline and treatment endpoint. Excluding their data from average calculations resulted in an overall average 1.41 rpm at baseline and 0.09 rpm at endpoint, yielding a 93.28% reduction.

**Table 3:** Average Baseline Rates, Response Rate Reduction across Treatment Phases, and Treatment Phase Descriptions

ID	Function Targeted	Design	A (RPM)	B (%RR)	C (%RR)	D (%RR)	E (%RR)	F (%RR)	Phase Descriptions
1	Auto	ABAB	4.24	89.39	-	-	-	-	B = MS + DR
2	Auto	ABCDADE	1.41	42.55	48.23	63.83	92.91	-	B = DR C = NCR + DR D = NCR + DR + RB E = NCR + DR + RB + VSTO
3	Auto	ABAB	1.36	82.60	53.62	79.39	87.48	96.32	B = DR C = DR + FCT D = DR + FCT + CS E = DR + FCT + CS + EXT F = DR + FCT + CS + BH
4	Auto	ABAB	0.38	100	-	-	-	-	B = DR + CS + FCT + EXT
5	Tan	ABAB	1.88	94.68	-	-	-	-	B = MS + DR
6	Tan	MBL	0.82	85.37	-	-	-	-	B = FCT + EXT
7	Tan	ABCAC	1.23	73.17	97.56	-	-	-	B = HH + FCT C = MS + HH + FCT + EXT
8	Tan	ABAB	1.96	95.92	-	-	-	-	B = HH + DR
9	Tan	ABABCD	2.43	99.84	81.48	98.77	-	-	B = NCR + RB C = NCR D = NCR + HH
10	Tan	ABABC	1.87	100	98.93	-	-	-	B = HH + FCT + EXT + RB C = NCR + MS + HH + FCT + Delay Tolerance + EXT + RB
11(1)	Tan	ABCDAD	0.20	0.00	0.00	60.00	-	-	B = EXT C = NCR + EXT D = NCR + MS + EXT + RB
11(2)	Attn	A_1_A_2_A_1_A_2_BA_2_BC	1.42^a^	69.01	97.18	-	-	-	A_1_ = BL in open common area A_2_ = BL in large room B = NCR + MS + FCT + EXT C = NCR + MS + FCT + EXT + RB
12	Tan, Auto	ABCAC	0.39	51.28	64.10	-	-	-	B = HH + DR + EXT + RB C = HH + DR + VSTO
13	Tan, Auto, Attn	ABABCAC	1.43	94.41	88.11	-	-	-	B = FCT + EXT C = NCR + FCT + EXT
14(1)	Attn	ABA	0.01	100	-	-	-	-	B = FCT + EXT
14(2)	Tan (objects)	ABAB	0.00	0.00	-0.40	-	-	-	B = MS + FCT + EXT C = NCR + MS + FCT + EXT
14(3)	Tan (activity)	ABAB	0.11	95.45	91.82	-	-	-	B = MS + FCT + EXT C = MS + FCT + SST + EXT

^a^ This value represents average responses per minute across two baseline phases (A_1_ average RPM = 1.83, A_2_ average RPM = 1.11)

*Note:* %RR = Percentage response reduction; Attn = Attention; Auto = automatic; BH = Baskethold; BL = Baseline phase; CS = Chained schedule; DR = Differential reinforcement; EXT + Extinction; FCT = Functional Communication Teaching; HH = Hand holding; NCR = Noncontingent reinforcement; MS = Multiple schedules; RB = Response blocking; RPM = Responses per minute; SST = Social skills teaching; Tan = Tangible; VSTO = Visual Screen Time-Out

When examining mean reduction for treatments grouped by function targeted, on average, treatments targeting an automatic function resulted in a 91.88% response reduction. Treatments targeting a tangible function resulted in a 95.50% response reduction. Treatments targeting an attention function resulted in a 97.20%. Treatments targeting multiple functions resulted in an average reduction of 82.97%.

Fourteen treatments achieved the targeted goal of an 80% or greater reduction in elopement attempts from baseline, and of these, 11 resulted in 90% or higher response reduction. It is important to note that one treatment, 14(1), did technically result in a 100% reduction, though this was from a very low (0.01 rpm) baseline rate. Likewise, treatment 14(2) showed 0.00 rpm in baseline and low rates in treatment, resulting in an increased rate of 0.40%. Txs 11(1) and 12 did not achieve the targeted goal, achieving 60.00% and 64.10% response reductions, respectively. Tx 11(1) observed three elevated datapoints during the final treatment phase, which increased overall rate calculation, though the majority (88%) of data points during the final treatment phase showed 0 rpm. Tx 12, which used both reinforcement and punishment procedures, began 2 weeks prior to P12’s discharge, and only five treatment sessions in the final treatment phase were conducted. Thus, the treatment team had insufficient time to conduct a thorough evaluation. Considering the relationship between function on treatment outcomes, all automatic treatments, seven out of nine tangible treatments, all attention treatments, and one out of two treatments targeting multiple functions resulted in an 80% reduction in elopement attempts. For exemplar graphs of individual treatment evaluations, see Figs. 2 and 3. All remaining treatment graphs can be found in the *Supplemental Materials*.

Regarding treatment components used, the most frequently used treatment components were extinction (*n* = 10), FCT (*n* = 10), multiple schedules of reinforcement (*n* = 8), and NCR (n = 7). Treatments most frequently used reinforcement strategies (*n* = 16) followed by antecedent-based (*n* = 10) and instructional methods (*n* = 10), and punishment strategies least frequently (*n* = 5).

### Generality of Treatment Effects

Data measuring generality of treatment effects in community-based settings were available for the nine treatments. Six of the remaining treatments tested generality of effects by implementing the treatment with novel adults and in novel locations on the inpatient unit. Tx 14(1), which failed to effectively evoke elopement during baseline or treatment phases, was the only treatment that was not generalized. Table 4 lists the number of sessions testing treatment generality, mean rpm of elopement across sessions, and the locations and format of sessions. Across treatments that conducted community-based sessions, rates of elopement remained low (0.00–0.14 rpm) except in Tx 1, where elopement equaled 0.32 rpm.

**Table 4:** Generality Outcomes across Treatments

ID	Nr of Sessions	RPM (M)	Generalization Locations/Formats
1	3	0.32	Community outings
2	36	0.10	Community outings, home, and with parents as implementers
3	-	-	None (to novel adults and common areas of unit only)
4	-	-	None (to novel adults and common areas of unit only)
5	-	-	None (to novel adults and common areas of unit only)
6	6	0.02	Community outings
7	-	-	None (to novel adults and common areas of unit only)
8	10	0.06	All community generalization with parents (n = 6) or novel adults (n = 4) as implementers
9	-	-	None (to novel adults and common areas of unit only)
10	-	-	None (to novel adults and common areas of unit only)
11(1)	-	-	None (to novel adults and common areas of unit only)
11(2)	1	0.00	Community outing to novel building
12	All	0.14^a^	All sessions conducted off-unit around hospital and in community
13	2	0.00	Community outings with novel adults as implementer
14(1)	-	-	None (to novel adults and common areas of unit only)
14(2)	19	0.00	Community outings with mother as implementer
14(3)	11	0.06	Community outings with mother as implementer

^a^ This reflects average responses per minute for the final treatment phase

## Discussion

The aim of this study was to characterize behavioral, function-based treatments that were systematically developed for elopement in children with autism who exhibited high rates of challenging behavior. Conducting assessments and treatment evaluations for elopement in controlled settings offered the advantage of being able to use creative approaches to the systematic execution of FAs and treatments evaluations, while maintaining sufficient environmental control to avoid risk of harm. By describing treatment efficacy, individual treatment components, and procedures used to test the generality of treatments, these findings can help guide assessment and treatment decisions of community-based practitioners for this difficult-to-treat topography of behavior.

When considering functions of elopement, access to preferred objects or activities was identified most frequently as a singular or at least one maintaining function, followed by automatic reinforcement. It should be noted that no cases of elopement were identified as being maintained by escape from common stressors (e.g., schoolwork, chores). These results suggested that children may in some cases be better described as “running to” something or “running for fun” as opposed to “running away.” Anderson et al. (2012) noted similar findings from a large-scale parent survey, which indicated that the most common reason parents thought their child eloped was that they “simply enjoy running and/or exploring” (56%), “to reach a place he/she enjoys” (36%), and/or “to pursue his/her special topic” (30%). In that study, escape from anxiety-provoking situations or sensory stimuli were reported in 34% and 30% of cases, respectively, and were more common in children with milder autism symptoms. Likewise, in a review of research in this area, Boyle & Adamson (2017) found that the most common functions were to access items or activities (60%) or attention (55%), but only 25% engaged in elopement to escape from demands. It is important to note that the maintaining effect of attention may have been underrepresented in this study, as for most cases reviewed, a duration-based FA procedure was used that required participant retrieval following elopement, and therefore likely introduced some confounding effects of attention. Although there is no current consensus on how to best control for the delivery of attention during resetting procedures, previous studies (e.g., Blowers et al., 2020) have developed procedures to control for attention effects during elopement FAs which may serve as an important model to follow.

Fourteen (82%) treatment evaluations met the treatment goal of an 80% or higher reduction in elopement attempts. The three treatments that did not meet the 80% reduction goal were limited by reduced time of the patient’s hospital admission, a failure to successfully evoke elopement during baseline, and a low baseline rate of elopement. Potential explanations for the zero rates of elopement observed in Txs 14(1) and 14(2) suggested that their FAs may have inaccurately identified function, or the treatment conditions failed to successfully evoke elopement for some unidentified reason. Tangible treatments demonstrated greatest consistency in showing zero rates of elopement near treatment endpoint, whereas treatments targeting an automatic function witnessed greater variability in responding at treatment endpoint. This is likely because treatments targeting tangible functions may more readily be able to establish stimulus control over elopement, whereas stimulus control is more challenging to achieve when behavior has an automatic function. Treatments targeting multiple functions had the lowest average response reduction, suggesting that future elopement treatments may be more effective in targeting functions individually. The findings of the current study are consistent with Call et al. (2017) in showing large reductions in elopement resulting from behavioral treatment, and consistent with Call et al. (2017) and Scheithauer et al. (2020) in demonstrating the need for a multicomponent treatment package that included antecedent and reinforcement strategies.

Examining treatment components, packages most frequently used extinction, FCT, multiple schedules of reinforcement, and DR. In several treatments, multiple schedules were signaled using red and green cards, which is a helpful approach to clearly communicating the availability and unavailability of reinforcers such as running opportunities or tangibles. Although a valuable tool, future clinicians should carefully consider choices for discriminative stimuli to account for potential color blindness or color sensitivity of autistic children and discrimination skills or use stimuli that can be discriminated based on features other than color. Schedule thinning was used during several applications of DR and multiple schedules of reinforcement once stable, low rates of elopement were observed. Due to limitations inherent in these treatments being tested in a hospital setting, schedules of reinforcement were thinned to varying intervals, again with the expectation that community-based therapists would continue to thin schedules following participants’ discharges.

The efficacy of nine treatments was tested outside of the hospital unit in the community, with caregivers, teachers, community-based therapists, novel adults, novel buildings, and in the participant’s home. The remaining eight treatments were generalized from the treatment team to novel staff and locations on the treatment unit. Across treatments, teams worked with participants’ families, community-based therapists, and teachers to support the continued implementation of treatment plans post-discharge by providing written behavior plans and follow-up consultation to families and community treatment teams upon request. Elopement rates remained low across treatments except in Tx 1, which is likely due to the small number of sessions that were conducted in the community.

The three treatments that used procedures identified through a stimulus avoidance assessment (i.e., visual screen time-out or baskethold) all targeted an automatic function, which emphasizes the challenge of successfully treating elopement with this function. Treatment teams placed several measures in place to ensure the ethical and short-term use of these procedures. Procedures were not evaluated without discussion with and consent from participants’ legally authorized representatives. These techniques were intentionally very brief (i.e., 1 min or less) and closely monitored by supervising BCBAs and treatment unit supervisors. Supervising BCBAs provided recommendations to community-based teams on how to gradually fade these procedures postdischarge once stable, low rates were observed in the community. Treatment teams considering such punishment procedures should use them only as last resort options after antecedent and reinforcement-based procedures in combination have been found insufficient in reducing elopement to safe levels via data collection. As demonstrated in this CCCS, they should not be considered without consent from legally authorized representatives, rigorous monitoring of correct implementation and fading procedures from qualified BCBAs with expertise in severe challenging behavior (Pokorski & Barton, 2021), and a clear fading plan.

Specialized training in severe behavior is necessary to replicate assessment and treatment procedures outlined in this CCCS safely. Therapists with expertise in elopement treatments should be consulted if treatment teams do not possess expertise in severe behavior; otherwise, clients should be referred to clinics that demonstrate such expertise. The current sample of participants and their families having resorted to intensive inpatient treatment for severe challenging behavior including elopement raised the importance of the increased availability of community-based interventions to target elopement. Unfortunately, one study examining predictors of access to elopement prevention strategies among autistic pediatric patients found that only 45% of their patient population used ABA therapy, and that access to ABA was predicted by family socioeconomic status (estimated based on maternal educational level; Pereira-Smith et al., 2019). Therefore, in addition to there being a need to build stronger evidence for treatments that eliminate elopement, there is a need to increase accessibility of qualified ABA providers and related treatments to families from underserved communities.

This study had several strengths, including the use of a clinical cohort that was identified retrospectively so that relationships between behavioral functions could be identified without specific intervention bias. Further, this study avoided demonstrating selection bias by including all cases who received a targeted assessment and treatment for elopement and whose treatment design allowed for the demonstration of experimental control, thereby maximizing the opportunity for generality of findings (Hagopian, 2020). Data were collected using a reliable and detailed system that allowed for multiple behavioral topographies and environmental variables to be measured. Standard data collection practices on the inpatient unit included the use of a controlled clinical environment with trained therapists and observers who could implement standardized protocols consistently with high integrity and while ensuring patient safety. This is typically more challenging to achieve in community contexts given the dangers associated with elopement.

Concerning limitations, elopement was evaluated for the most part in a controlled clinical setting, and limited data were available assessing generality of treatment effects. Future evaluations of elopement treatments would need to test treatment effects in less controlled settings for longer periods. Although the current dataset benefitted from demonstrating high IOA, it lacked treatment fidelity data, as limitations in resources within the inpatient unit precluded having enough staffing to collect these data. Although historically not the standard for CCCS, future CCCS should consider reporting treatment fidelity data to demonstrate the validity of findings. Finally, the use of multielement, duration-based FAs meant that contingent on elopement, the relevant reinforcer assessed in each condition was delivered for a predetermined reinforcement period, after which the opportunity to elope was reset. Sessions were conducted in open areas or involving the use of two rooms to allow participants the opportunity to safely elope without blocking attempts. This “retrieval” method allowed therapists to minimize the necessity to physically intervene with participants and has been evaluated in several studies examining the function of elopement (e.g., Boyle & Adamson, 2017). Studies that have compared FA outcomes with assessment methods that did versus did not involve retrieval have found similar outcomes across methods (e.g., Lehardy et al., 2013). However, this should still be noted as a limitation, as the synthesis of contingencies in this type of FA likely still posed problems with interpretation of the outcomes because it necessarily involved the consistent delivery of attention after each condition. In community-based settings, treatment teams may find latency and trial-based FAs to be an easier and more effective approach to assessing elopement function. Trial-based FAs minimize the need for resetting procedures during an FA session, whereas assessing latency to elopement over rate may provide a more valid assessment of the potency of tested reinforcers (Kamlowsky et al., 2021; Lambert, 2016).

Future research on elopement should continue to explore safe and effective methods for assessing and reducing elopement, particularly in populations who are more challenging to support, and that are accessible to participants across communities. Research should also focus on advancing the field by identifying strategies for preventing elopement when it is too challenging to block (i.e., in large children and adults). Example treatments identified in this analysis that relied on non-blocking-based strategies warrant replication in larger samples. Additional research should explore how to target elopement maintained by other possible functions identified through parent surveys in Anderson et al. (2012), such as escape from anxiety or aversive sensory stimuli that may contribute to sensory meltdowns. Finally, given the prevalence of this issue within the autism community and the inherent risk that elopement presents, there is a critical need to educate the general community about elopement prevention and response strategies as further ways to mitigate risk.

## Supplementary Information

Below is the link to the electronic supplementary material.

Supplementary file1 (PDF 245 kb)

## Data Availability

The data that support the findings of this study are available from the Kennedy Krieger Institute Neurobehavioral Unit but restrictions apply to the availability of these data, which were used under license for the current study, and so are not publicly available. Data are however available from the authors upon reasonable request and with permission of the Kennedy Krieger Institute.

## Footnotes

1 Note that in Frank-Crawford et al. (2024), the dependent variable was the latency to elopement. For the current study, we transformed latency into rate by dividing the number of times the participant eloped during the session by the total session duration (in min).
