# Supplementary material for: A Retrospective Consecutive Controlled Case Series Analysis of the Assessment and Treatment of Elopement in Children with Autism in an Inpatient Setting
Source: Behav Anal Pract. 2024 Oct 24;18(4):903–20. doi: 10.1007/s40617-024-00979-1 (PMC12779866; doi:10.1007/s40617-024-00979-1)

Figure 4

Treatment 3 Evaluation

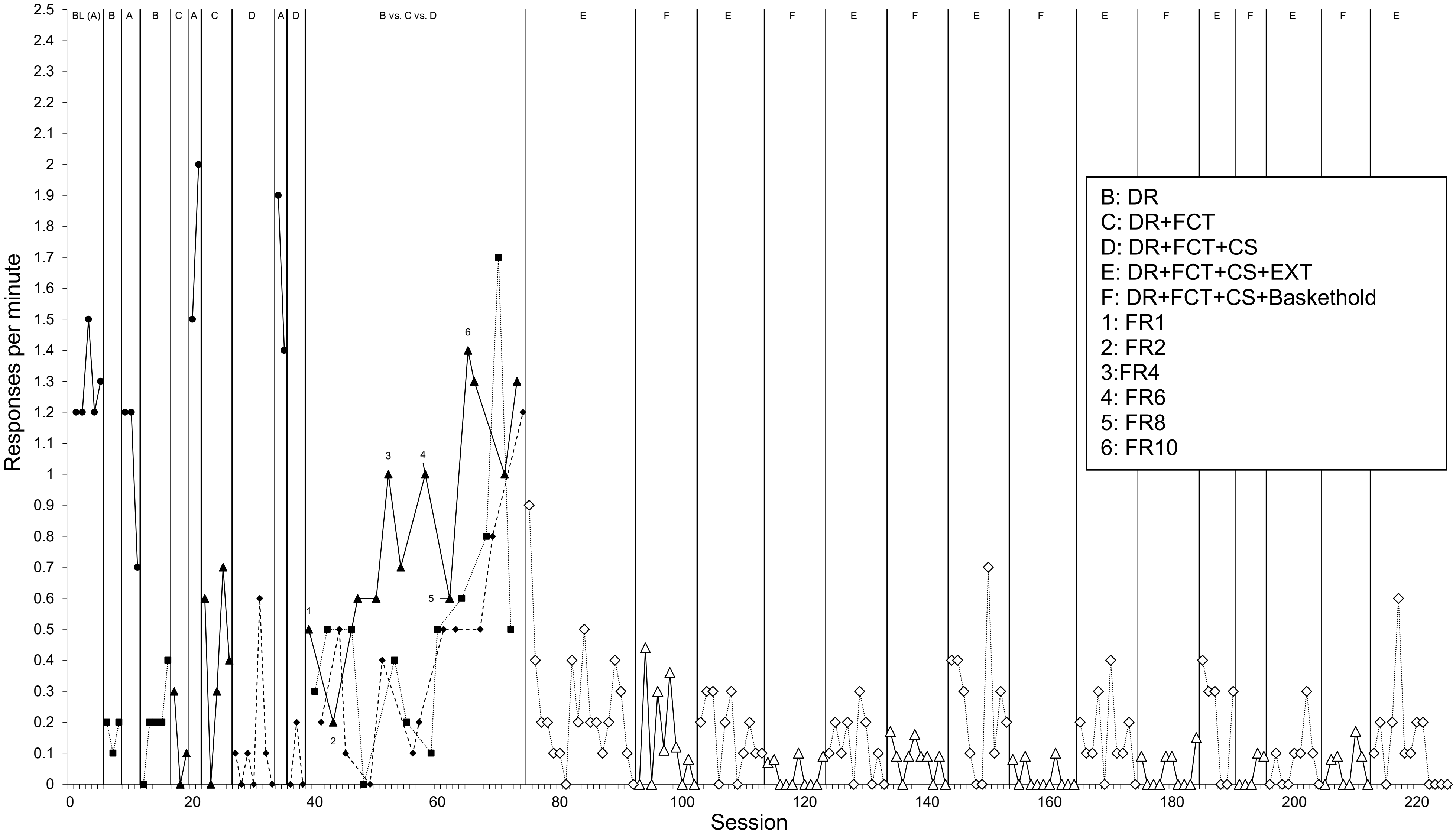

Figure 5

Treatment 4 Evaluation

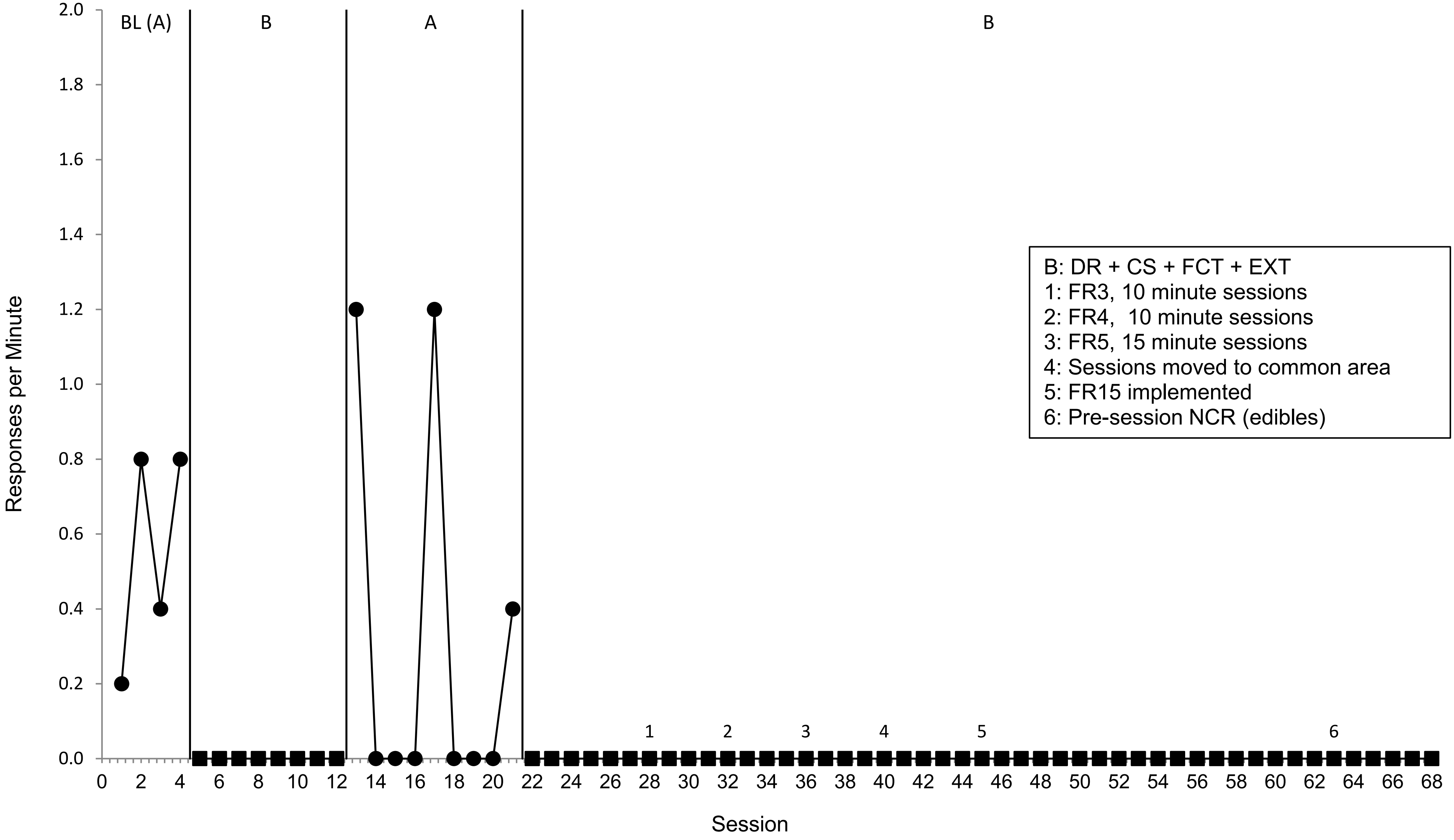

Figure 6

Treatment 5 Evaluation

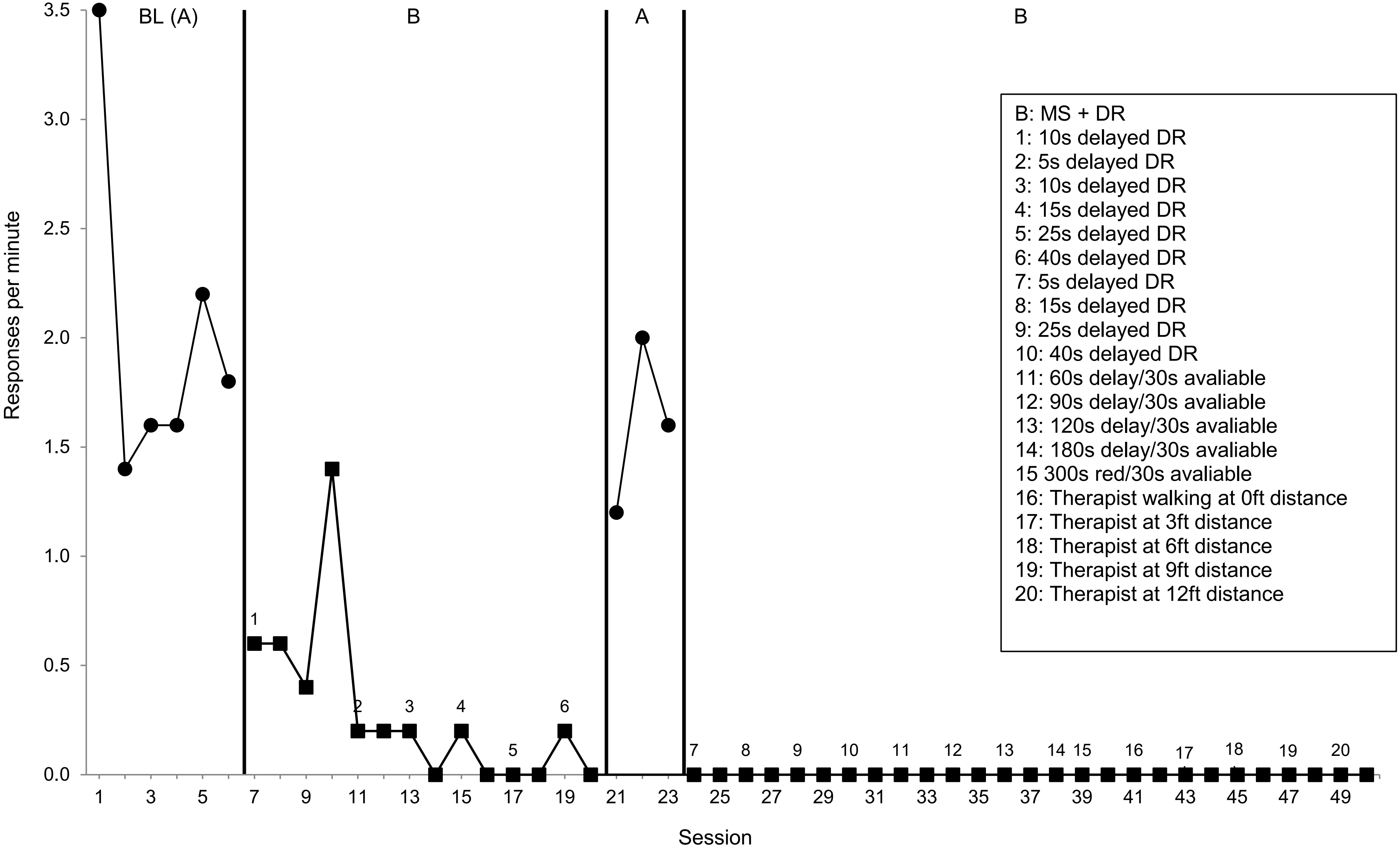

Figure 7

Treatment 6 Evaluation

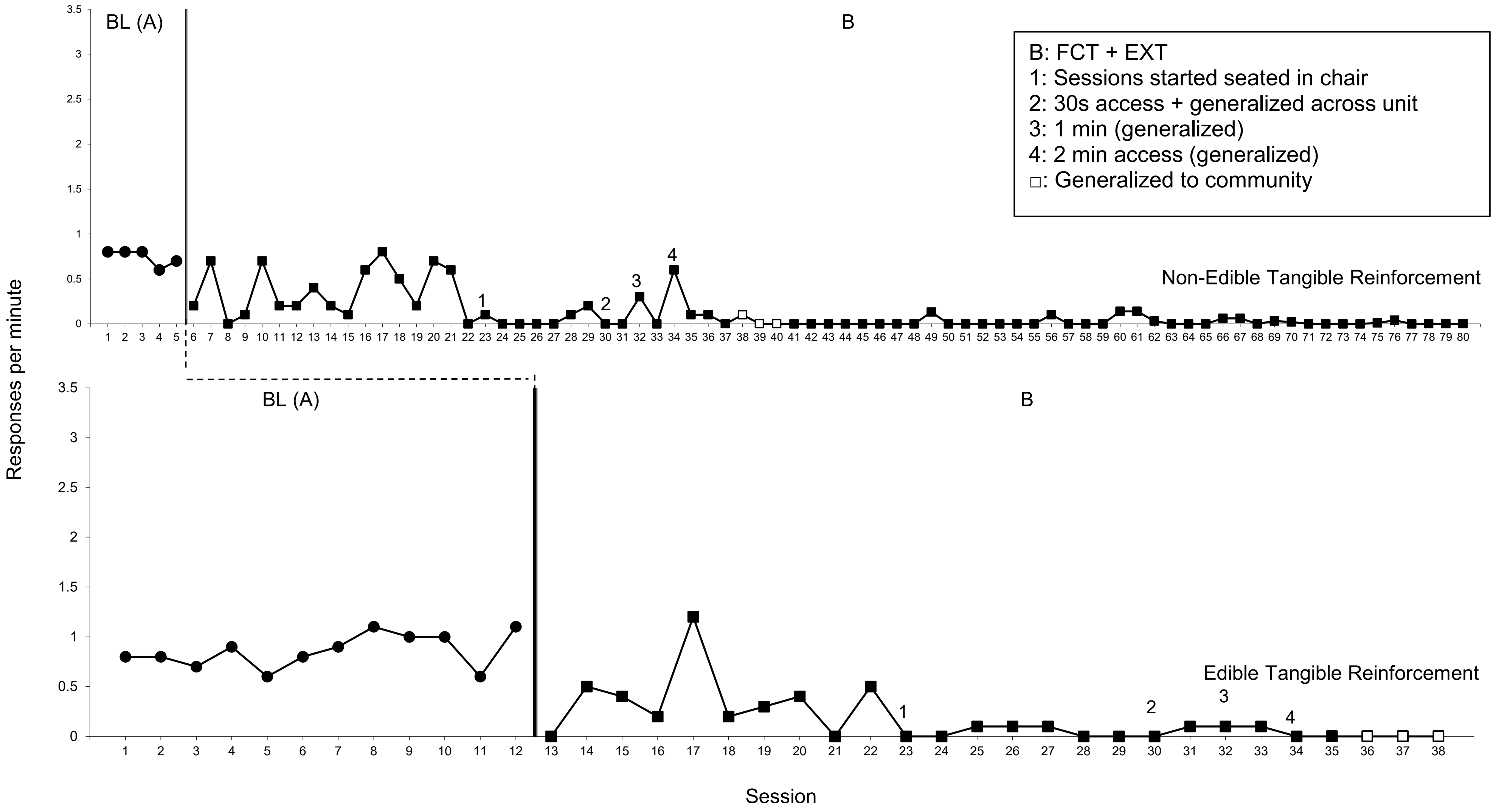

Figure 8

Treatment 7 Evaluation

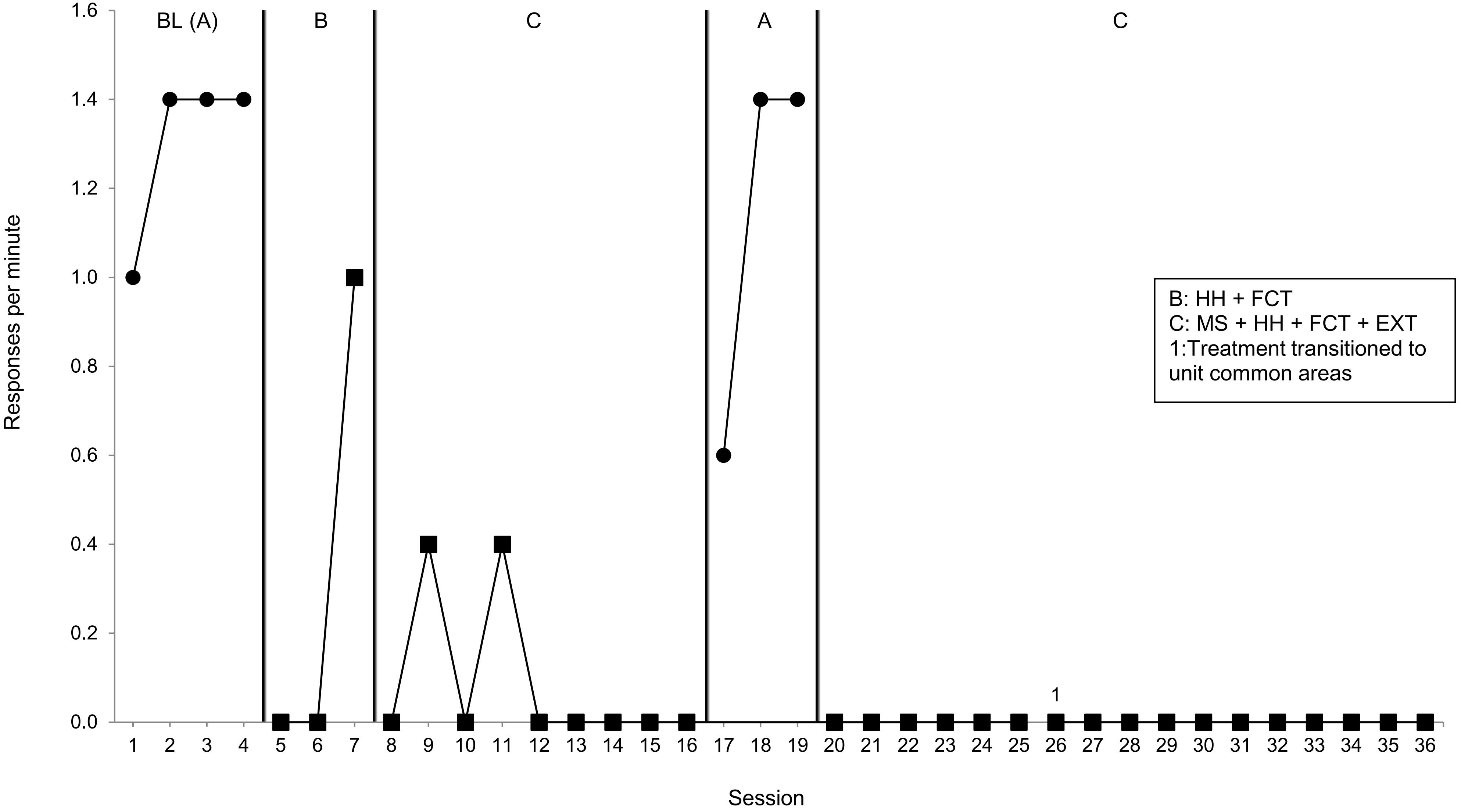

Figure 9

Treatment 8 Evaluation

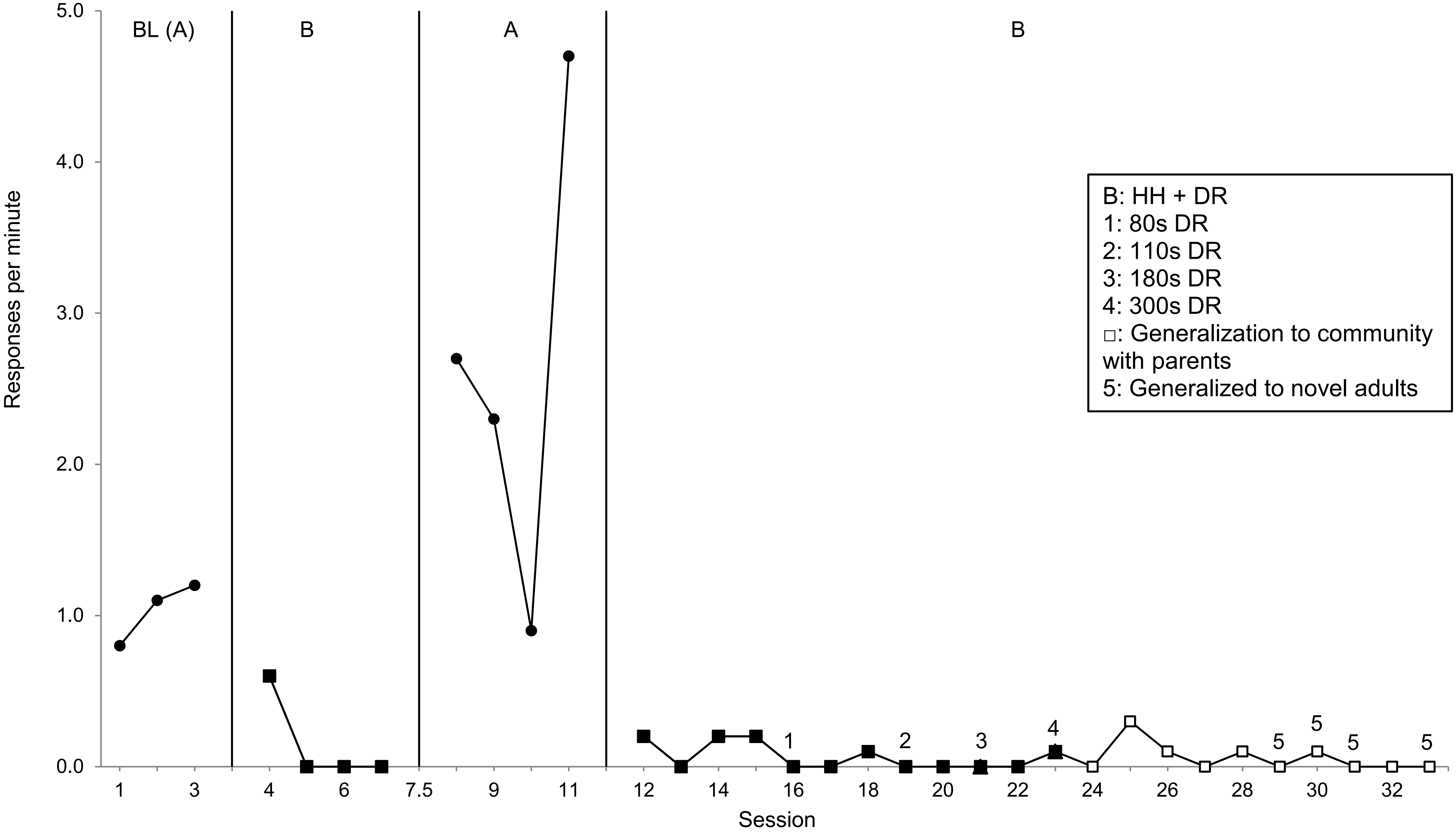

Figure 10

Treatment 9 Evaluation

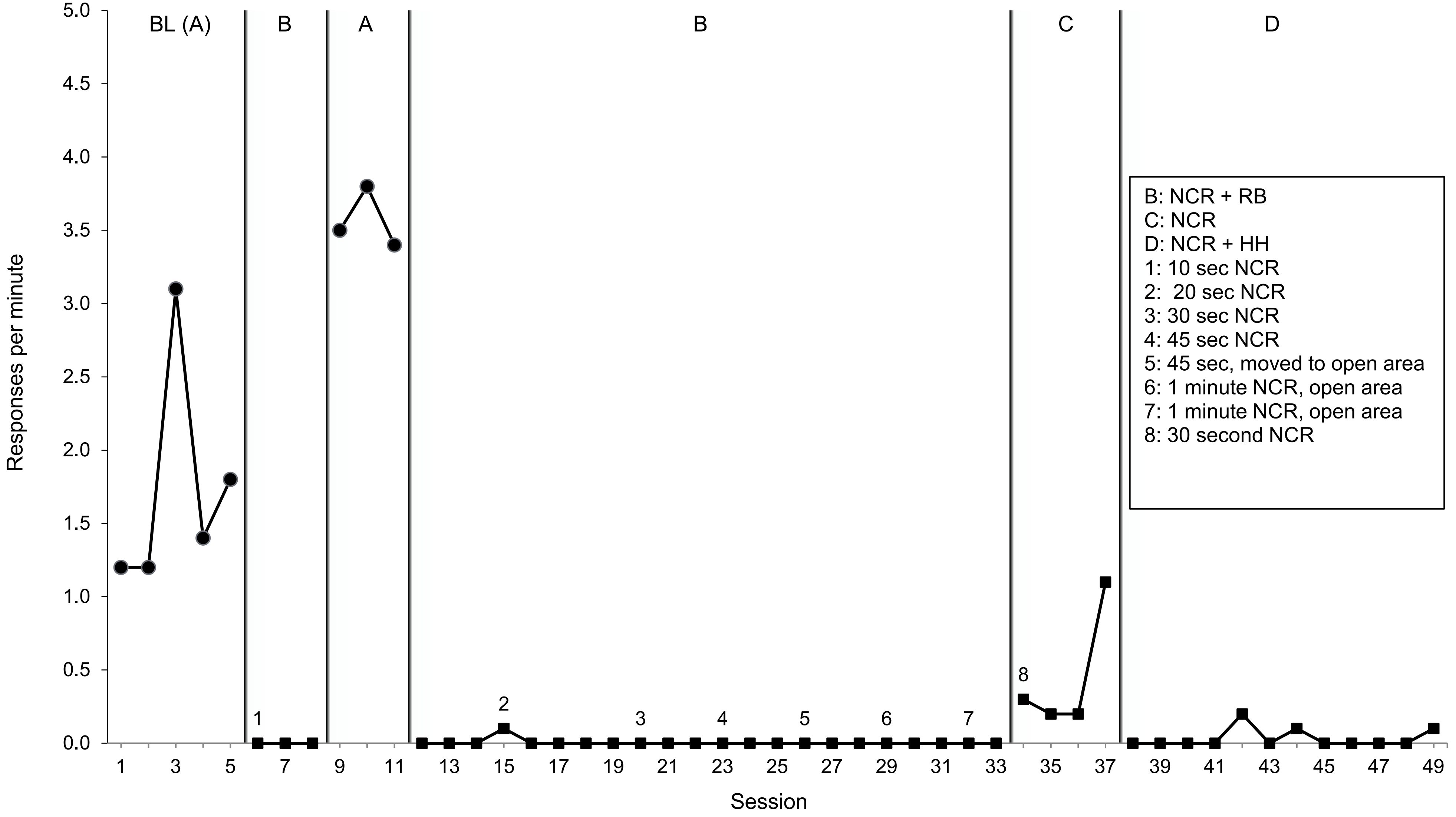

Figure 11

Treatment 10 Evaluation

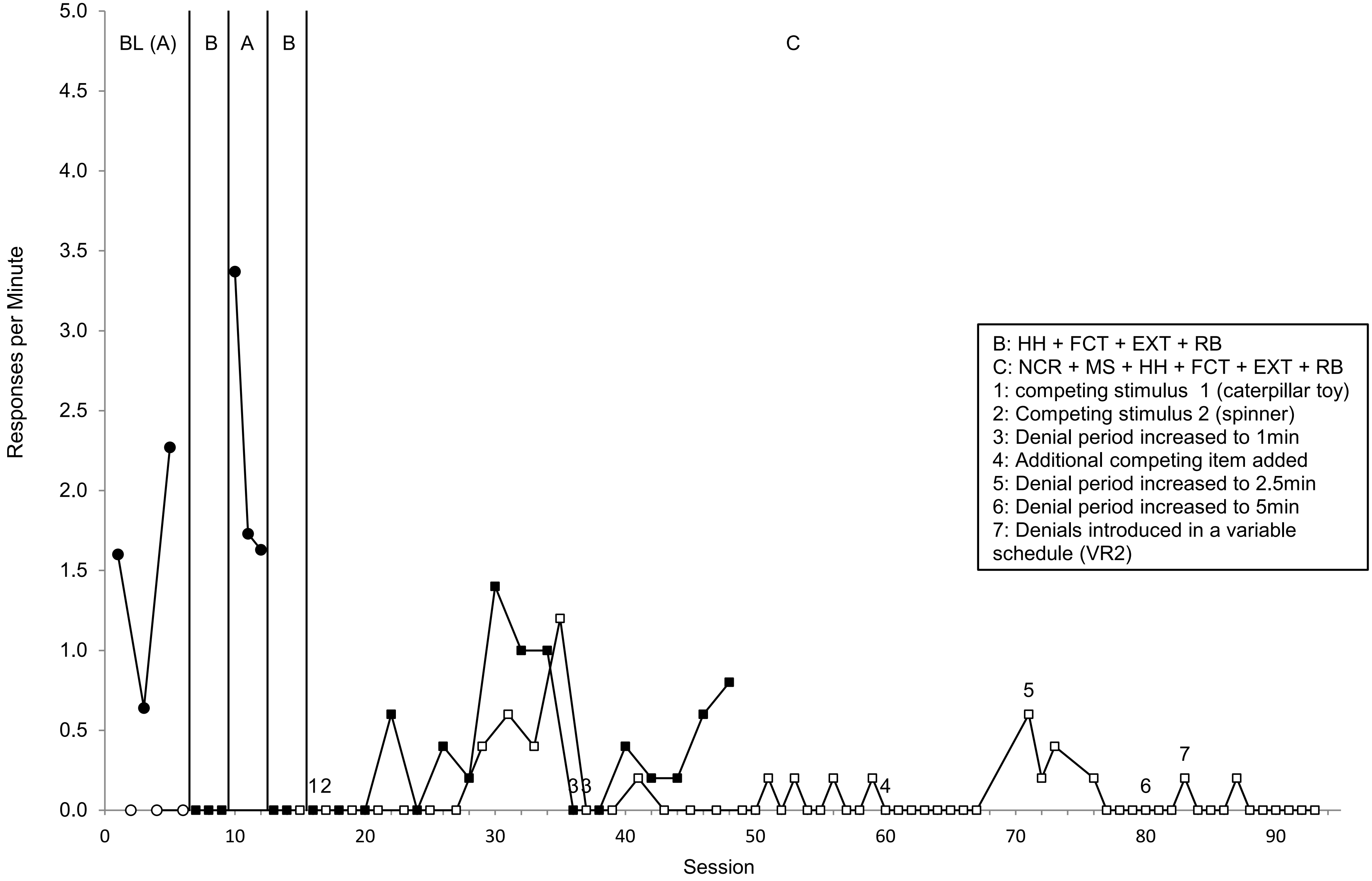

Figure 12

Treatment 11(1) Evaluation

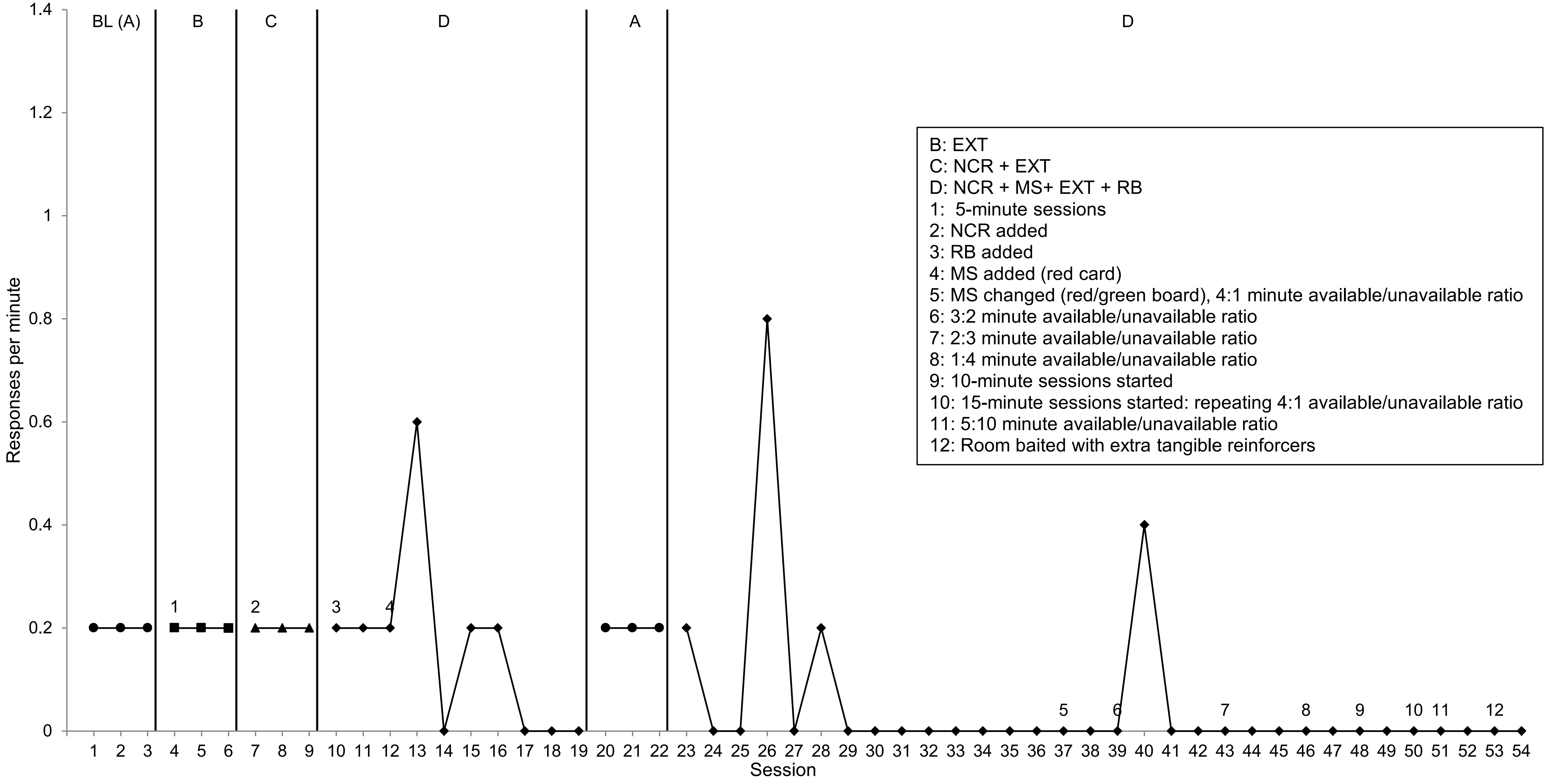

Figure 13

Treatment 11(2) Evaluation

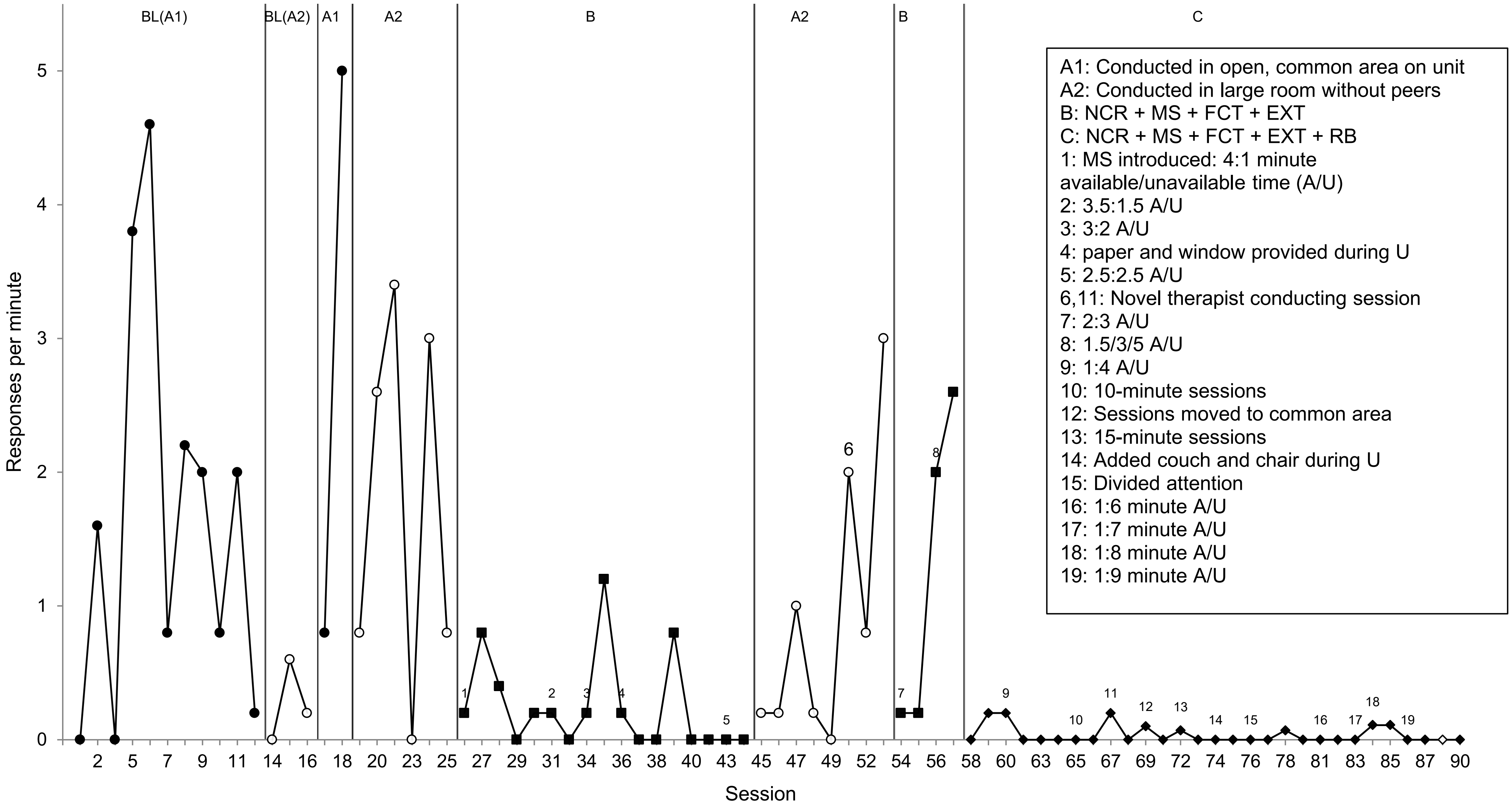

Figure 14

Treatment 12 Evaluation

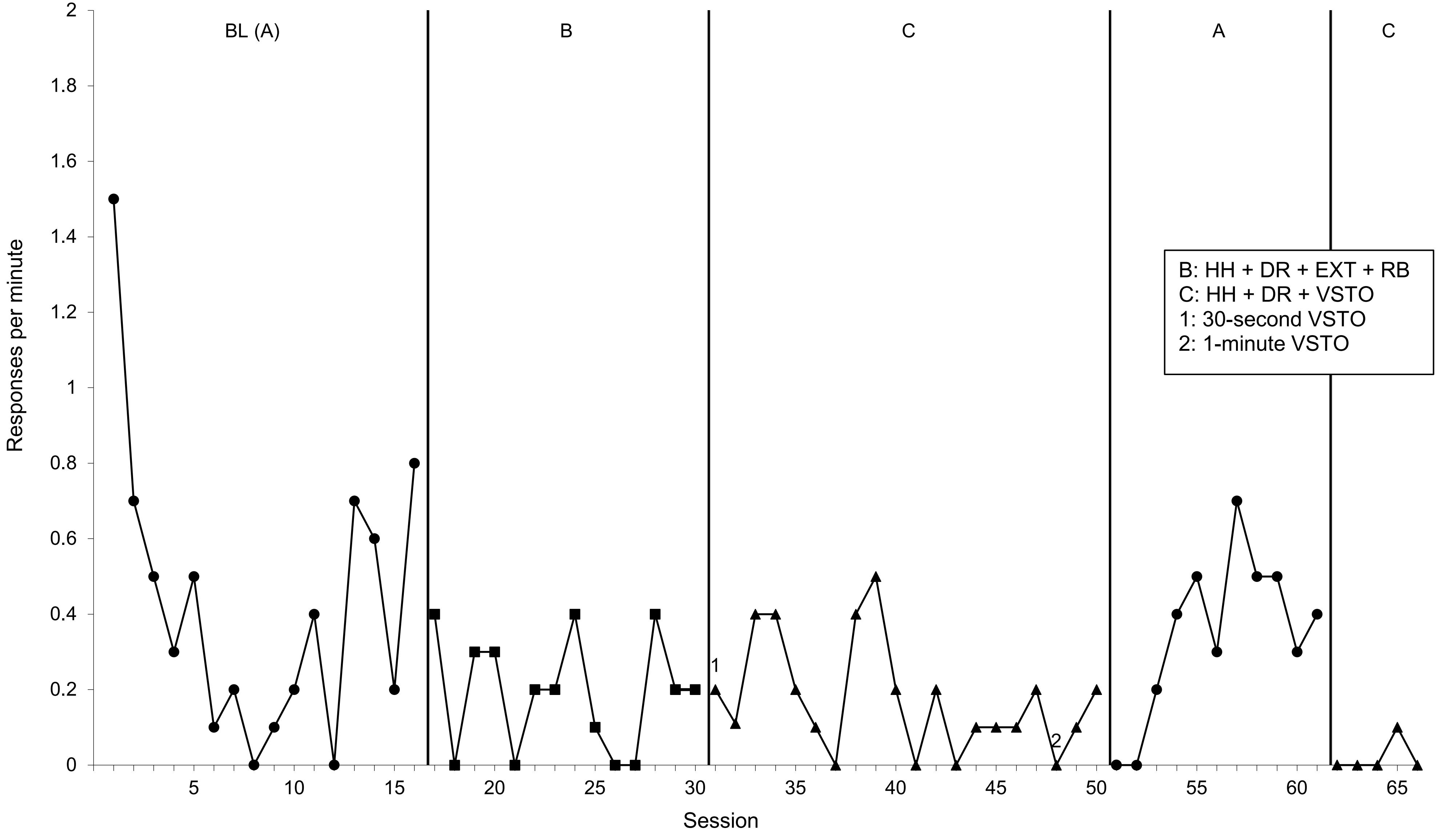

Figure 15

Treatment 13 Evaluation

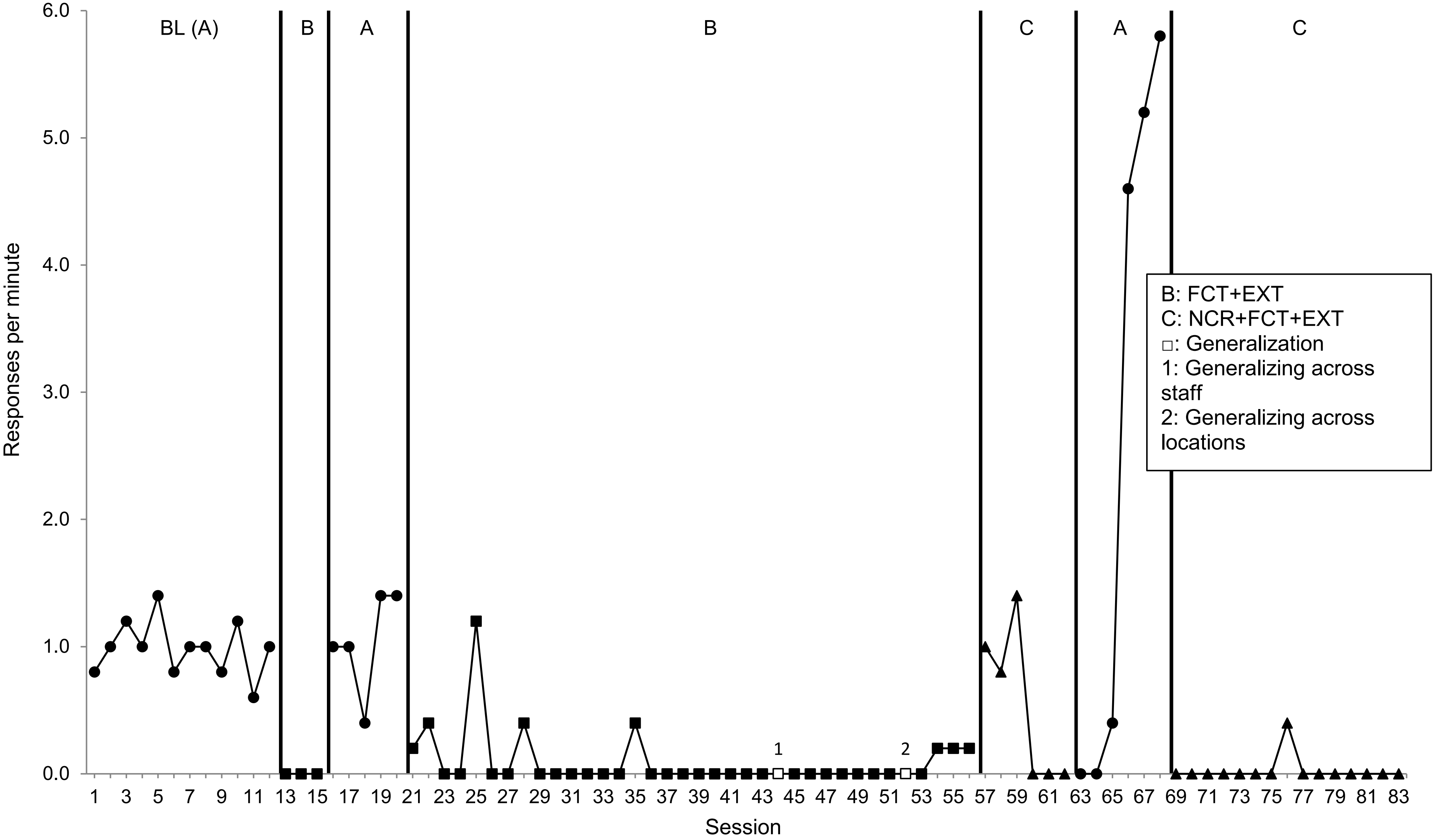

Figure 16

Treatments 14(1-3) Evaluations

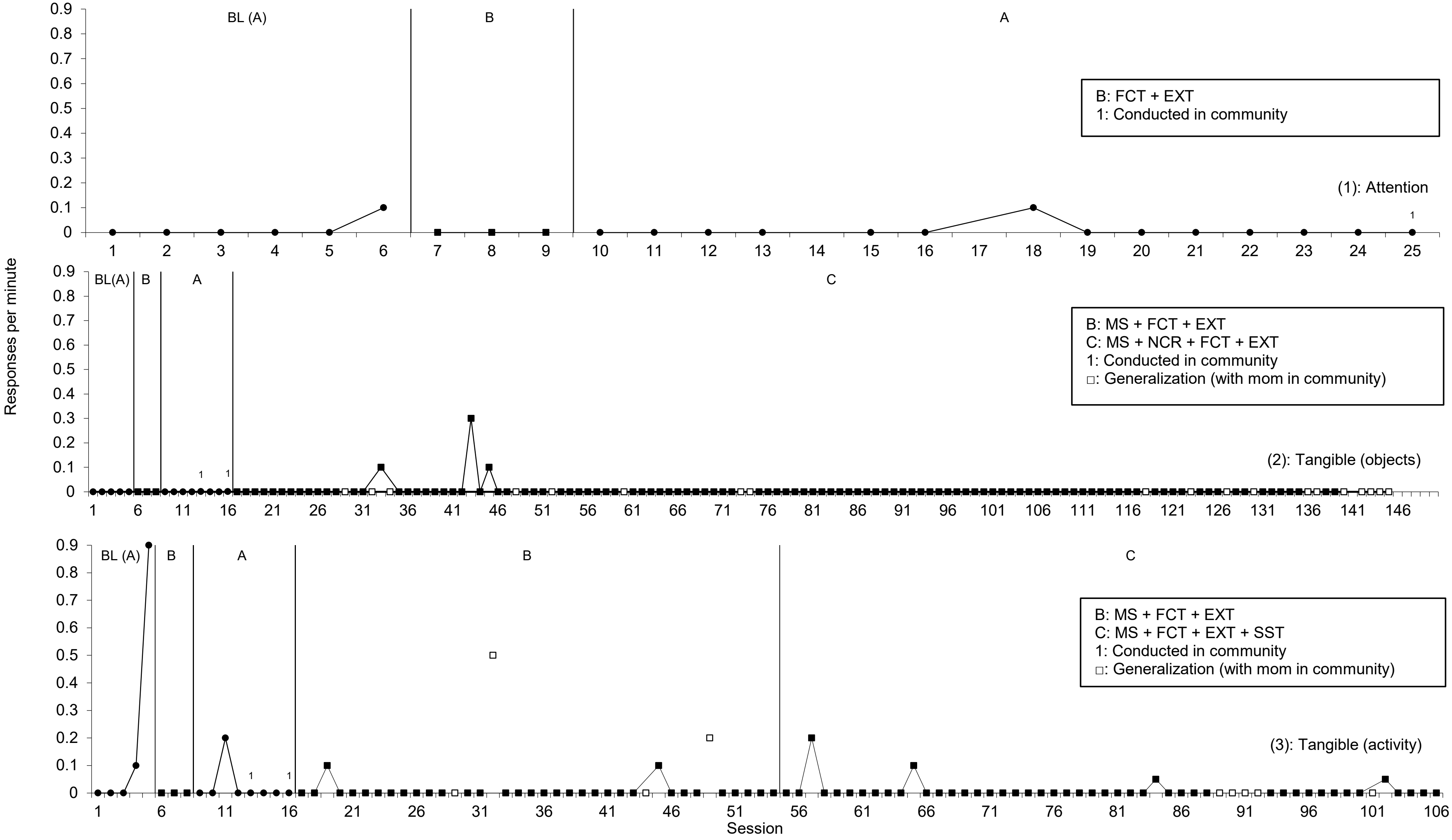

Figure 17

Graphical Exemplar of Functional Analysis Results for Participant 7

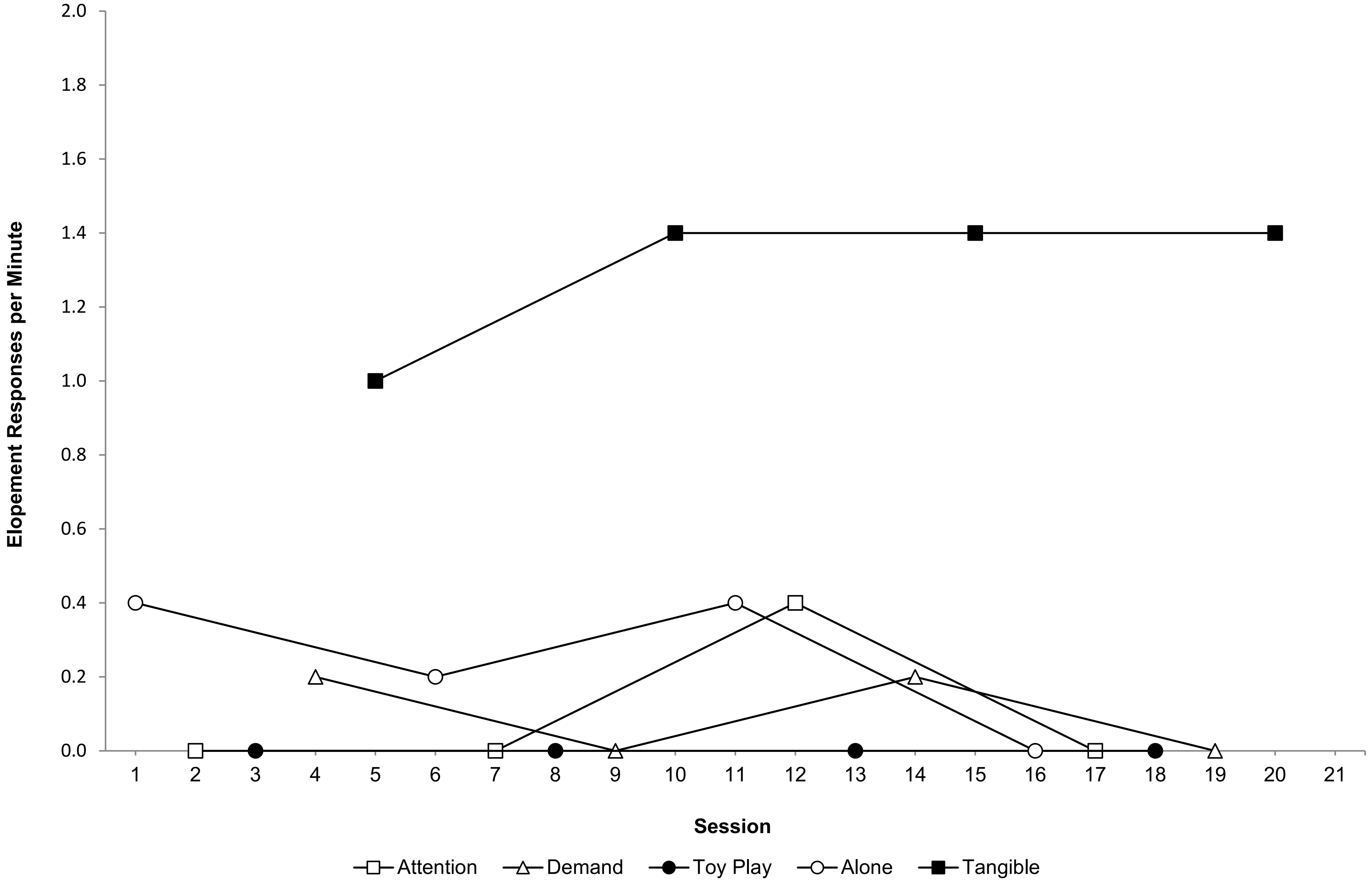

Supplement: Supplementary file 1 — Supplementary file1 (PDF 245 kb) [file 40617_2024_979_MOESM1_ESM.pdf]
